# Impact of Rotavirus Vaccines on Gastroenteritis Hospitalizations in Western Australia: A Time-series Analysis

**DOI:** 10.2188/jea.JE20200066

**Published:** 2021-08-05

**Authors:** Parveen Fathima, Mark A Jones, Hannah C Moore, Christopher C Blyth, Robyn A Gibbs, Thomas L Snelling

**Affiliations:** 1 Wesfarmers Centre of Vaccines and Infectious Diseases, Telethon Kids Institute, The University of Western Australia, Perth, Western Australia, Australia; 2 School of Medicine, University of Western Australia, Perth, Western Australia, Australia; 3 Department of Infectious Diseases, Perth Children’s Hospital, Perth, Western Australia, Australia; 4 Department of Microbiology, PathWest Laboratory Medicine WA, Perth Children’s Hospital, Perth, Western Australia, Australia; 5 Communicable Disease Control Directorate, Department of Health, Perth, Western Australia, Australia; 6 Menzies School of Health Research and Charles Darwin University, Casuarina, Northern Territory, Australia; 7 School of Public Health, Curtin University, Perth, Western Australia, Australia

**Keywords:** rotavirus vaccine, acute gastroenteritis, vaccine impact, hospitalizations, time-series

## Abstract

**Background:**

Rotavirus vaccination was introduced into the Australian National Immunisation Program in mid-2007. We aimed to assess the impact of the rotavirus vaccination program on the burden of hospitalizations associated with all-cause acute gastroenteritis (including rotavirus gastroenteritis and non-rotavirus gastroenteritis) in the Aboriginal and non-Aboriginal population in Western Australia.

**Methods:**

We identified all hospital records, between July 2004 and June 2012, with a discharge diagnosis code for all-cause gastroenteritis. Age-specific hospitalization rates for rotavirus and non-rotavirus acute gastroenteritis before and after the introduction of the rotavirus vaccination program were compared. Interrupted time-series models were used to examine differences in the annual trends of all-cause gastroenteritis hospitalization between the two periods.

**Results:**

Between July 2004 and June 2012, there were a total of 106,974 all-cause gastroenteritis-coded hospitalizations (1,381 rotavirus-coded [15% among Aboriginal] and 105,593 non-rotavirus gastroenteritis-coded [7% among Aboriginal]). Following rotavirus vaccination introduction, significant reductions in rotavirus-coded hospitalization rates were observed in all children aged <5 years (up to 79% among non-Aboriginal and up to 66% among Aboriginal). Among adults aged ≥65 years, rotavirus-coded hospitalizations were 89% (95% confidence interval, 16–187%) higher in the rotavirus vaccination program period. The time-series analysis suggested reductions in all-cause gastroenteritis hospitalizations in the post-vaccination period among both vaccinated and unvaccinated (age-ineligible) children, with increases observed in adults aged ≥45 years.

**Conclusions:**

Rotavirus vaccination has been associated with a significant decline in gastroenteritis hospitalizations among children. The increase in the elderly requires further evaluation, including assessment of the cost-benefits of rotavirus vaccination in this population.

## INTRODUCTION

Gastroenteritis is a leading cause of morbidity in young children worldwide in both developed and developing countries.^1^ In Western Australia, gastroenteritis is the second most common infection-related cause of hospitalization in young children after acute lower respiratory infections.^2^ Gastroenteritis hospitalization rates among Aboriginal and Torres Strait Islander (henceforth referred to as Aboriginal) children in Western Australia have been estimated to be nearly 5 times higher than that in non-Aboriginal children.^3^

Globally, rotavirus is the most common cause of severe dehydrating gastroenteritis in young children.^4^ Currently, two live attenuated oral rotavirus vaccines—a two-dose monovalent human rotavirus vaccine RV1 (Rotarix; GlaxoSmithKline Biologicals, Rixensart, Belgium), and a three-dose pentavalent human-bovine reassortant vaccine RV5 (RotaTeq; Merck Vaccines, Whitehouse Station, NJ, USA) have been licensed for use in Australia. Since July 2007, rotavirus vaccination has been included in Australia’s National Immunisation Program (NIP) for all children born on or after May 1, 2007.^5^ The programme in Western Australia provided RV1 at ages 2 and 4 months from July 2007, and then switched to RV5 at ages 2, 4, and 6 months from July 2009. In pre- and post-licensure studies, both RV1 and RV5 have demonstrated efficacy and effectiveness against severe rotavirus gastroenteritis and all-cause gastroenteritis requiring medical attention among children in both developed and developing countries.^6^

Our group has previously reported on the temporal trends of gastroenteritis-coded hospitalizations in Western Australian-born children aged less than 5 years, in particular noting the much greater incidence of hospitalizations among Aboriginal children.^3^ However, to our knowledge, the impact of the rotavirus vaccination program on the burden and epidemiology of acute gastroenteritis (including rotavirus gastroenteritis) in the Western Australian population (in both adults and children) has not been quantified. This study aimed to describe the impact of the rotavirus vaccination program on the burden of hospitalizations associated with all-cause acute gastroenteritis (including rotavirus gastroenteritis and non-rotavirus acute gastroenteritis) in the Aboriginal and non-Aboriginal population in Western Australia. Also, we conducted an interrupted time-series analysis to examine differences in the annual trends in hospital admissions for gastroenteritis between the pre-rotavirus and rotavirus vaccination periods.

## METHODS

Western Australia is Australia’s largest state geographically, with a total land area of more than 2.5 million km^2^. As of June 2016, approximately 3.9% of Western Australia’s population of 2.6 million identified as Aboriginal.^7^ Hospitalization data were sourced from the Western Australian Hospital Morbidity Data Collection (HMDC), which contains information on all inpatient admissions from public and private hospitals across Western Australia.^8^ The information includes socio-demographic details, dates of admission and separation (discharge) and discharge diagnosis codes (principal diagnosis, co-diagnosis and up to 20 additional diagnosis codes) using the International Classification of Diseases and Related Health problems, Tenth Revision, Australian Modification (ICD-10-AM) coding system.^8^

The study outcome was all-cause acute gastroenteritis-coded hospitalizations. We included all hospital records having an ICD-10-AM diagnosis code for rotavirus (A08.0) or other non-rotavirus acute gastroenteritis (A01 to A09 [excluding A08.0] and K52.9) in the principal or additional diagnosis fields. Only admission and separation dates between July 2004 and June 2012 were included. Admissions from the same person having the same diagnosis code within 14 days of a previous admission were grouped together and classified as a single episode of illness. Aboriginal status of the individual was identified using the derived Aboriginal status provided by Western Australian Data linkage branch.^9^

### Statistical analysis

Using Australian Bureau of Statistics derived annual estimated resident population estimates (downloaded from the Rates Calculator version 9.5, Western Australian Department of Health), crude age-specific rates and crude incidence rate ratios (IRRs) for the periods prior to (July 2004–June 2007) and post (July 2007–June 2012) introduction of rotavirus onto the NIP were calculated. Estimates were calculated separately for rotavirus and non-rotavirus gastroenteritis hospitalizations for the different age groups among both Aboriginal and non-Aboriginal individuals. Exact 95% confidence intervals (CIs) and IRRs were calculated using EpiBasic (version 4; University of Aarhus, Nordre Ringgade, Denmark).

Interrupted time-series models were used to evaluate changes in the all-cause gastroenteritis-coded and rotavirus-coded hospitalization rates from July 1, 2004 through November 30, 2012. For the time series, January 1, 2007 through December 31, 2007 (6 months prior to and following the July 1, 2007 introduction of the rotavirus vaccination program) was considered as the vaccine rollout period, July 1, 2004 to December 31, 2006 was considered as the pre-rotavirus vaccination or pre-rollout period, and January 1, 2008 onwards as the post-rotavirus vaccination or post-rollout period. Hospitalization data for rotavirus and non-rotavirus gastroenteritis were combined to assess all-cause gastroenteritis in each age group. Also, due to low absolute monthly numbers of hospitalizations in the Aboriginal population, the time-series analysis was not stratified by Aboriginal status. As *post-hoc* analysis, we also modelled rotavirus-coded hospitalizations for the <12 months, 12–23 months, and 2-year age groups—the rotavirus data was too sparse to model for all age groups.

Our approach is based on published recommendations and example analyses for applying segmented regression in interrupted time series.^10^^–^^12^ We fitted an impact model to each series using the Generalized Linear Autoregressive Moving Average (GLARMA) framework.^13^ We adopted negative binomial distributional assumptions when the data were over-dispersed and Poisson distributional assumptions otherwise. Equation 1 below provides the specification of the linear predictor linear predictor.

**Equation 1**

$$y_{t}∼\text{NegBin}(\mu_{t},\phi ),\;\mu_{t}=n_{t}\theta_{t}$$

$$\begin{array}{rl} Log({\text{µ}}_{\text{t}}) & =\beta_{0}+\beta_{1}\;{\text{time}}_{\text{t}}+\beta_{2}\;{\text{time since rollout}}_{\text{t}}\\ & \;+\beta_{3}\;{\text{time post rollout}}_{\text{t}}+\beta_{4}I\;({\text{rollout stage}}_{\text{t}})\\ & \;+\beta_{5}I\;({\text{post rollout stage}}_{\text{t}})+\beta_{6}I\;({\text{winter}}_{\text{t}})\\ & \;+\beta_{7}I\;({\text{spring}}_{\text{t}})+\beta_{8}I\;({\text{summer}}_{\text{t}})\\ & \;+\log({\text{population estimate}}_{\text{t}}) \end{array}$$

Equation 1 assumes the data are in time order *t* = 1…*T* with

*y_t_* denoting the counts of hospitalizations,
μ*_t_* and ϕ the location and scale parameters,
*n_t_* being the exposure (population at time *t*),
θ*_t_* being modelled by the exponentiated linear predictor,
β*_k_* denoting the parameter for the *k^th^* regressor and
*I*( ) denoting indicator variables.

Equation 1 includes an intercept term and terms to model the pre-rollout, rollout and post-rollout level (vertical displacement from zero), a pre-intervention linear trend, a change in linear trend during the rollout period (all of 2007), a change in linear trend during the post-rollout stage, and changes in level during the winter, spring and summer seasons. When outbreaks were known to have occurred, we introduced an additional indicator variable to model them and we included autoregressive (AR) terms in the models if the residual diagnostics suggested temporal dependence in the auto-correlation function (ACF) plots. We visually assessed residual diagnostics including residual partial-autocorrelation and autocorrelation functions to see whether modelling assumptions had been violated. We conservatively retained the full specification for all series and did not undertake model reduction. That is, we assumed that the direct effects associated with the vaccine and the indirect effects associated with herd protection, would follow the impact model. In instances where we compared models, goodness-of-fit was evaluated using Akaike Information Criterion (AIC)^13^ and likelihood ratio tests (LRT). Further detail on the methods is provided in the supplementary document (eMaterials 1 and eMaterials 2).

### Ethical approvals

Ethical approvals were obtained from the Western Australian Department of Health Human Research Ethics Committee and the University of Western Australia. As per the request of the data custodians, individual table cell sizes of less than 5 have been suppressed.

## RESULTS

### Rotavirus-coded hospitalizations

Between July 2004 and June 2012, there were a total of 1,381 hospitalizations coded as rotavirus gastroenteritis, of which approximately 15% (*n* = 200) occurred among the Aboriginal population. Rotavirus-coded hospitalization rates were highest in children aged <24 months in both population groups in the pre-rotavirus vaccination and rotavirus vaccination period (Table 1). Comparing the two time periods, significant declines were observed in Aboriginal children aged <12 months (by 66%; 95% CI, 49–77%) and 12–23 months (by 57%; 95% CI, 24–76%; Table 1) in the rotavirus vaccination period. In the non-Aboriginal population, along with declines in these two age groups, declines were also observed among those aged 2–4 years (by 59%; 95% CI, 49–68%) in the rotavirus vaccine periods (Table 1). Also, although hospitalization rates in the elderly were relatively low, a significant increase in hospitalization rates in the rotavirus vaccination period was observed among those aged ≥65 years (IRR 1.89; 95% CI, 1.16–2.87) in the non-Aboriginal population (Table 1).

**Table 1.** Rotavirus-coded hospitalization rates per 1,000 population, incidence rate ratios before and after the introduction of rotavirus vaccination, by Aboriginal status and age group, in Western Australia July 2004–June 2012

**Age group**	**Aboriginal**					**Non-Aboriginal**				

	Jul ‘04–Jun ‘07		Jul ‘07–Jun ‘12		IRR (95% CI)^a^	Jul ‘04–Jun ‘07		Jul ‘07–Jun ‘12		IRR (95% CI)^a^
	
	*n*	Rate/1,000	*n*	Rate/1,000		*n*	Rate/1,000	*n*	Rate/1,000	
**<12 months**	74	15.47 (12.15, 19.42)	41	5.31 (3.81, 7.20)	0.34 (0.23, 0.51)	170	2.42 (2.07, 2.82)	109	0.75 (0.62, 0.91)	0.31 (0.24, 0.40)
**12–23 months**	33	6.64 (4.57, 9.33)	23	2.88 (1.82, 4.32)	0.43 (0.24, 0.76)	221	3.12 (2.72, 3.56)	92	0.65 (0.52, 0.79)	0.21 (0.16, 0.27)
**2–4 years**	10	0.64 (0.31, 1.18)	11	0.44 (0.22, 0.78)	0.68 (0.26, 1.79)	167	0.77 (0.66, 0.90)	128	0.31 (0.26, 0.37)	0.41 (0.32, 0.51)
**5–9 years**	<5	N/A	<5	N/A	N/A	39	0.10 (0.07, 0.14)	44	0.07 (0.05, 0.09)	0.65 (0.41, 1.02)
**10–19 years**	<5	N/A	<5	N/A	N/A	8	0.01 (0.00, 0.02)	18	0.01 (0.00, 0.02)	1.29 (0.53, 3.43)
**20–44 years**	<5	N/A	<5	N/A	N/A	10	0.00 (0.00, 0.01)	15	0.00 (0.00, 0.01)	0.81 (0.34, 2.01)
**45–64 years**	<5	N/A	<5	N/A	N/A	13	0.01 (0.00, 0.02)	28	0.01 (0.01, 0.01)	1.15 (0.58, 2.43)
**≥65 years**	<5	N/A	<5	N/A	N/A	27	0.04 (0.03, 0.06)	92	0.07 (0.06, 0.09)	1.89 (1.16, 2.87)

CI, confidence interval; IRR, incident rate ratio; N/A, not applicable.

^a^Incidence rate ratio of rotavirus vaccine period to pre-rotavirus vaccine period hospitalization rate.

### Non-rotavirus acute gastroenteritis-coded hospitalizations

A total of 105,593 hospitalizations coded as non-rotavirus acute gastroenteritis were identified between July 2004 and June 2012, of which approximately 7% (*n* = 6,975) occurred among the Aboriginal population. In both the Aboriginal and non-Aboriginal population, the highest rates for these hospitalizations were associated with children aged <24 months in both time periods followed by adults aged ≥65 years (Table 2). Compared to the pre-rotavirus vaccination period, significant declines were observed among Aboriginal children aged <24 months in the rotavirus vaccination period (Table 2). Among the non-Aboriginal population, significant declines were observed in those aged up to 9 years in the rotavirus vaccination period (Table 2). Similar to rotavirus-coded hospitalizations, an increase in non-rotavirus acute gastroenteritis-coded hospitalizations rates were observed among non-Aboriginal adult age groups in the period after the introduction of the rotavirus vaccination program.

**Table 2.** Non-rotaviral acute gastroenteritis-coded hospitalizations rates per 1,000 population, incidence rate before and after the introduction of rotavirus vaccination, by Aboriginal status and age group, in Western Australia July 2004–June 2012

**Age group**	**Aboriginal**					**Non-Aboriginal**				

	Jul ‘04–Jun ‘07		Jul ‘07–Jun ‘12		IRR (95% CI)^a^	Jul ‘04–Jun ‘07		Jul ‘07–Jun ‘12		IRR (95% CI)^a^
	
	*n*	Rate/1,000	*n*	Rate/1,000		*n*	Rate/1,000	*n*	Rate/1,000	
**<12 months**	551	115.18 (105.76, 125.21)	616	79.76 (73.59, 86.32)	0.69 (0.62, 0.78)	1,164	16.60 (15.66, 17.58)	1,664	11.45 (10.91, 12.02)	0.69 (0.64, 0.74)
**12–23 months**	516	103.84 (95.08, 113.20)	634	79.35 (73.29, 85.77)	0.76 (0.68, 0.86)	1,214	17.14 (16.19, 18.13)	1,367	9.62 (9.12, 10.15)	0.56 (0.52, 0.61)
**2–4 years**	307	19.63 (17.50, 21.96)	487	19.34 (17.66, 21.14)	0.99 (0.85, 1.14)	1,417	6.54 (6.20, 6.88)	1,730	4.22 (4.02, 4.42)	0.65 (0.60, 0.69)
**5–9 years**	122	4.56 (3.79, 5.45)	237	5.39 (4.72, 6.12)	1.18 (0.95, 1.48)	881	2.33 (2.18, 2.49)	1,197	1.81 (1.71, 1.92)	0.78 (0.71, 0.85)
**10–19 years**	139	2.94 (2.47, 3.47)	224	2.69 (2.35, 3.06)	0.91 (0.74, 1.14)	1,374	1.68 (1.59, 1.77)	2,436	1.71 (1.64, 1.78)	1.02 (0.95, 1.09)
**20–44 years**	554	7.22 (6.63, 7.85)	886	6.47 (6.05, 6.91)	0.89 (0.80, 1.00)	8,023	3.78 (3.70, 3.86)	15,989	4.06 (4.00, 4.12)	1.07 (1.05, 1.10)
**45–64 years**	360	13.29 (11.95, 14.74)	830	15.66 (14.61, 16.76)	1.18 (1.04, 1.34)	7,527	5.11 (4.99, 5.22)	16,030	5.82 (5.73, 5.92)	1.14 (1.11, 1.17)
**≥65 years**	187	31.69 (27.31, 36.57)	325	28.94 (25.87, 32.26)	0.91 (0.76, 1.10)	11,866	16.94 (16.64, 17.25)	24,739	18.64 (18.41, 18.87)	1.10 (1.08, 1.12)

CI, confidence interval; IRR, incident rate ratio.

^a^Incidence rate ratio of rotavirus vaccine period to pre-rotavirus vaccine period hospitalization rate.

### Time-series analysis of all-cause gastroenteritis

**Figure 1.**
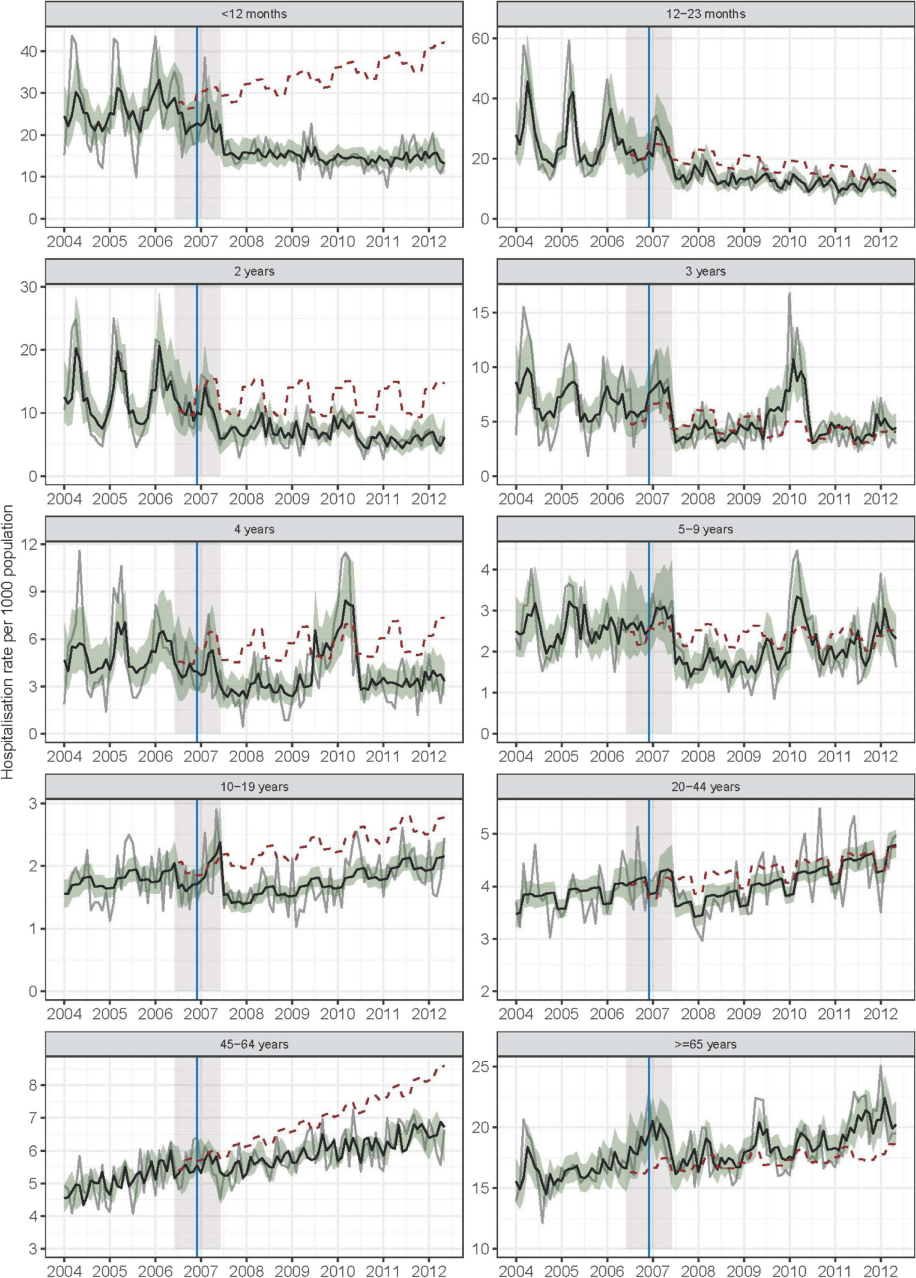
Observed (grey) and modelled (black) monthly all-cause gastroenteritis coded hospitalizations per 1,000 population 2004–2012 by age class. Grey solid line shows observed all-cause gastroenteritis-coded hospitalizations. Black solid line shows modelled all-cause gastroenteritis-coded hospitalizations. Red dashed lines show hypothetical values predicted from the model for the rollout and post-rollout periods under the assumption that vaccination had not been introduced. Blue vertical shows the date vaccine was introduced (1 July 2007). Year x-axis tick marks at June 30.

Figure 1 shows the observed and modelled monthly series of all-cause gastroenteritis-coded hospitalizations per 1,000 population. The figure also shows hypothetical values predicted from the model for the rollout and post-rollout periods under the assumption that vaccination had not been introduced. Over the study period, there were a total of 106,974 all-cause gastroenteritis-coded hospitalizations, giving an overall age-standardized rate of 6.27 per 1,000 population. Crude hospitalization rates over the study period were highest in the <12 months age group (19.3 per 1,000 population) followed by the 12–23 months and ≥65 years age groups (both 18.2 per 1,000 population).

eTable 1 and eTable 2 provides parameter estimates from the models fitted to the data for each age group. Broadly, the series associated with the <12 months, 12–23 months, and 2 years age groups had similar features (ie, these series had higher variability in the pre-rollout period than in the post-rollout period), suggested weak trends, and showed positive autocorrelation with AR terms (*P* ≤ 0.001). Additionally, both the 12–23 months and 2 years age group models showed seasonal effects with uplifts in the hospitalization rates during the winter and spring seasons (eTable 1). While none of these three age group models had significant pre-rollout trend terms or post-rollout changes in trend (*P* ≥ 0.05), the <12 months and 2 years age group series showed statistically significant reductions in the post-rollout level relative to the pre-rollout period (eTable 1). Specifically, based on the data in the pre- and post-rollout periods, the parameter estimates for the change in level during the post-rollout period implied hospitalization rate reductions of 54% (95% CI, 29–71%; *P* < 0.001) in the <12 months, 36% (95% CI, −10 to 63%; *P* = 0.10) in the 12–23 months and 43% (95% CI, 3–66%; *P* = 0.04) in the 2 years age groups (eTable 1).

The all-cause gastroenteritis time series for the 3, 4, and 5–9 years age groups also shared similar characteristics to each other, with a notable pronounced peak in hospitalization rates in 2010 in those aged 3 and 4 years. Similar to the younger age cohorts, the pre-rollout periods had higher variability and higher mean hospitalization rates than the post-rollout periods and any trends that were present in the data were weak and hard to discern visually. None of the models as specified by Equation 1 suggested a change in level nor trends in the pre-rollout, rollout, or post-rollout period. However, compared to the pre-rollout period, the estimates for the change in level suggested hospitalization rate reductions of 36% (95% CI, −33 to 59%) among those aged 3 years, 62% (95% CI, −2 to 86%) among those aged 4 years, and 38% (95% CI, −4 to 63%) in those aged 5–9 years in the post-rollout period.

For the older children and adults in the 10–19, 20–44, and 45–64 years age groups, visual inspection suggested mild positive trends over the entire study period. Qualitatively, seasonality was less apparent as were level shifts between the pre-rollout and post-rollout periods. The 10–19 and the 20–44 years age groups did not show any residual temporal correlation (Durbin Watson test on residuals with *P*-values 0.92 and 0.35, respectively). Relative to the pre-rollout rate, the model estimates for the 10–19 years age group did suggest a reduction of 29% (95% CI, 9–44%) in the post-rollout hospitalization rate. We also note that seasonality was suggested by the data (*P*-value <0.01 for LRT for comparison of model fitted per Equation 1 relative to model fitted without a seasonal term). The model for the 45–64 years age group suggested a positive trend across the whole study period with a rate ratio of 1.01 (95% CI, 1.00–1.01) corresponding to a 1% increase in the hospitalization rate per month. However, the parameter that described the change in the pre-rollout trend in the post-rollout period had a rate ratio of 0.99 (95% CI, 0.99–1), which mostly offset the growth associated with the global trend term. Finally, among adults aged ≥65 years, the variability in this series was not as extreme as in the younger age groups and we did not find any evidence of seasonality. However, compared to the pre-rollout period, the estimate for the change in level suggested an instantaneous increase in hospitalization rates of 7% (95% CI, −11 to 30%) in the post-rollout period relative to the pre-intervention period and rates have continued increase through to 2012.

In the post-hoc analysis, although the point estimates suggested reductions in rotavirus-coded hospitalization rates in the post-rollout period, none of the time-series models as specified by Equation 1 showed strong trends or level shifts (eTable 2). We caution over-interpretation of this result as the data were sparse. These models also showed seasonal effects consistent with the pattern in the all-cause gastroenteritis series. Also, distinct spikes in hospitalization rates were observed among those aged >12 months. Time-series plots for all age groups for all-cause gastroenteritis-coded and rotavirus-coded hospitalizations are provided in eMaterials 3.

## DISCUSSION

Using total population-level hospitalization data, this ecological study has described the impact of the publicly funded rotavirus vaccination program on the temporal trends of both rotavirus-coded and non-rotavirus acute gastroenteritis-coded hospitalizations in Western Australia over an 8-year period. In this study, following the introduction of the vaccination program in mid-2007, significant reductions of up to 79% in rotavirus-coded hospitalization rates were observed in all non-Aboriginal children aged <5 years whereas, among the Aboriginal population, decline in hospitalization rates were only observed among children aged <2 years (up to 66%). The magnitude of these observed declines are similar to declines observed in other countries with low child mortality wherein hospitalizations and emergency department visits due to laboratory-confirmed rotavirus gastroenteritis declined by a median of 71% following the introduction of infant rotavirus vaccination program.^14^

In the period following rotavirus vaccination program introduction, significant reductions (of up to 44%) in non-rotavirus acute gastroenteritis-coded hospitalizations were also observed in children age-eligible for vaccination. Similar declines have been noted in other settings in Australia and elsewhere.^14^^–^^19^ This suggests that many episodes of rotavirus-related hospitalizations are assigned non-specific gastroenteritis diagnostic codes.^20^ An Australian study has shown that only a third of all hospitalizations that tested positive for rotavirus had a rotavirus-specific diagnostic code and only 62% of all all-cause gastroenteritis-coded hospitalizations that tested positive for rotavirus had a rotavirus-specific code, with no significant differences noted in the sensitivity and specificity of the coding in the pre and post-vaccine periods.^21^

In the time-series analysis, reductions in all-cause gastroenteritis-coded hospitalizations were observed after 2007 with the introduction of the rotavirus vaccination program, even among children who were not age-eligible to be vaccinated. This suggests an indirect protective effect of the vaccination program among unvaccinated children (ie, herd immunity), similar to those reported in other settings in Australia and other countries with universal rotavirus vaccination programs.^16^^,^^18^^,^^22^^–^^24^ A distinct increase in all-cause gastroenteritis- and rotavirus-coded hospitalization rates was observed in 2010. Rotavirus disease, like many infectious diseases, exhibits annual/seasonal cycles and studies have demonstrated an increase in the interepidemic period following the introduction of rotavirus vaccination programs.^25^^–^^27^ In 2010, it appears that the increase was confined to older children, suggesting this occurred in those too old to have been eligible for vaccination and who may have escaped natural infection in their earlier years due to the vaccine program.

Compared to the pre-rotavirus vaccine era, all-cause gastroenteritis rates were higher in the rotavirus vaccination program period among adults. Notifications for several non-rotavirus gastroenteritis have shown a steady increase in Western Australia since 2007, especially notifications for non-typhoidal salmonella relating to foodborne outbreaks caused by *Salmonella typhimurium* and *Salmonella enteritidis*.^28^ Among adults aged ≥65 years, rotavirus-coded hospitalizations were 1.89 times higher in the rotavirus vaccination program period. Increase in rotavirus-related hospitalization in those aged ≥65 years following introduction of rotavirus vaccination has been observed at a national level in Australia,^16^^,^^29^ but this finding contrasts with studies elsewhere which have shown indirect effects among adults.^30^^,^^31^ As with children, reduction in rotavirus circulation in the community following vaccine introduction could have resulted in minimal exposure to the virus leading to decreased immune boosting against the virus in this population. In a randomized controlled trial, RV5 has been demonstrated to be safe and immunogenic in healthy elderly adults aged 65–80 years.^32^ Keeping in mind the burden of rotavirus disease in the elderly, the value and cost-benefit of rotavirus vaccination in this population needs to be evaluated.

The strength of this study is that it is based on a large comprehensive population-based analysis of 8 years of hospitalization data spanning pre- and post-rotavirus vaccination periods. This enabled us to analyze the age-specific burden of rotavirus-coded and non-rotavirus acute gastroenteritis-coded hospitalizations prior to and after the introduction of rotavirus vaccination. Also, because we have reliable data on Aboriginal status, we were able to analyze hospitalization rates separately for Aboriginal and non-Aboriginal children. An important limitation of our study is that we lacked information on individual immunization status and therefore, the impact of rotavirus vaccination on the vaccinated and unvaccinated cohort could not be elicited. Also, we relied on discharge diagnosis codes to identify rotavirus-coded hospitalizations and information on the temporal changes in testing practices for rotavirus in the hospital setting in Western Australia over the study period was not available. This could have underestimated the true burden of rotavirus-related hospitalization in the population.

In conclusion, our findings demonstrate substantial reductions in rotavirus-specific and all-cause gastroenteritis-coded hospitalizations in children in Western Australia following the introduction of the rotavirus vaccination program. However, despite the implementation of a vaccine program with demonstrated herd immunity in children, this study did not find evidence of herd immunity among adults; our results showed a significant increase in rotavirus-coded and all-cause gastroenteritis-coded hospitalizations among adults aged ≥65 years denoting the susceptibility of this population to rotavirus. This increase in the elderly need further research to determine whether this is due to a real increase in disease with inadequate herd protection from the infant program or due to temporal changes in diagnostics practices. Continued population-level surveillance is warranted to assess the impact of rotavirus vaccination on the epidemiology of age-specific acute gastroenteritis across different age-groups and subpopulations.

## APPENDIX A. SUPPLEMENTARY DATA

eMaterials 1. Modelling approach

eMaterials 2. A narrative description of fitting the timeseries for a specific age-class

eMaterials 3. Timeseries and seasonality plots for all the age-classes >12 months

eTable 1. Parameter estimates from the GLARMA models fitted to the acute gastroenteritis-coded hospitalisations

eTable 2. Parameter estimates from the GLARMA models fitted to the rotavirus-coded hospitalisations
