# Supplementary material for: Impact of Rotavirus Vaccines on Gastroenteritis Hospitalizations in Western Australia: A Time-series Analysis
Source: J Epidemiol. 2021 Aug 5;31(8):480–6. doi: 10.2188/jea.JE20200066 (PMC8275440; doi:10.2188/jea.JE20200066)
Supplement: Supplementary file 1 [file je-31-480-s001.pdf]

eMaterials 1. Modelling approach

We outline further details regarding the modelling approach used for the interrupted time series analysis.

We assume an impact model whereby both the pre-intervention trend and level would change following the rollout of rotavirus vaccination program (all of 2007) and post-rollout (from 2008 to the end of the dataset) period. This follows standard recommendations provided in the literature [1]. We adopt a segmented negative binomial distributional assumption when the data were over-dispersed, but Poisson distributional assumptions otherwise. The linear predictor includes an intercept, terms for the pre-intervention, rollout and post-rollout trends (the latter two specified as a change from the pre-intervention trend), terms for the stage of the intervention (reference level being pre-intervention, then rollout followed by post-rollout) and terms for seasonality (Winter, Spring, Summer) relative to Autumn, see Equation 1 below. Population size (*exposure* in the language of Generalised Linear models [GLMs]) was included as an offset variable to convert the outcome into a rate and adjust for any changes in population over time.

We use the Generalised Linear Autoregressive Moving Average (GLARMA) framework to fit the data. GLARMA models are observation-driven state-space models that extend GLMs and allow explicit modelling of temporal correlation via the inclusion of autoregressive and moving average terms in the model [6]. GLARMA models can be fit using the glarma function from the R glarma package. Briefly, we outline this model class. Following [6], let y_t for $t = 1..T$ where T be the number of observations in the discrete response series and $\boldsymbol{x_t}$ be the K regression vectors. Let $\mathcal{F}_t = \{\Upsilon_s: s < t, x_t: s \leq t\}$ denote the set of past information on the response and past and present information on the regressors. We take the distribution of Υ_s conditional on $\mathcal{F}_t$ to be of the exponential family form:

$$f(y_t|W_t) = \exp\{y_t W_t - a_t b(W_t) + c_t\}$$

with a_t and c_t sequences of constants dependent on y_t and the state variable W_t a function of the elements in $\mathcal{F}_t$. Generally, the state vector is linear in the covariates:

$$W_t = x_t^T \beta + Z_t$$

with the form of Z_t best thought of as the linear predictor of a stationary invertible ARMA (autoregressive moving average) process with driving noise e_t

$$Z_t = \sum_{i=1}^{p} \phi_i (Z_{t-i} + e_{t-i}) + \sum_{i=1}^{q} \theta_i e_{t-i}$$

As outlined in the manuscript we take y_t as the count of hospitalisations for all-cause gastroenteritis or rotavirus specific hospitalisation and represent $x_t^T \beta$ as the impact model as detailed in Equation 1. We initially forego modelling temporal correlation as we prefer to be guided by residual diagnostics to determine whether serial dependence is present in the data. When we exclude ARMA terms, the fitted models degenerate to their GLM counterparts in that the parameter estimates and inference will be identical. In cases where residual correlation is apparent in the residual auto-correlation function (ACF) and partial auto-correlation function (PACF) plots, we apply heuristics [8] to interpret the order of the implied auto regressive (AR) and moving average (MA) process and refit the model with the additional ARMA terms. Using this approach, we can better account for

variability in the data under a more parsimonious specification that includes explicit modelling of temporal dependence.

Equation 1 below provides the impact model specification of the $x_t^T\beta$ component of the state vector W_t (assumes time ordered data).

Equation 1

$$y_t \sim \text{NegBin}(\mu_t, \phi) \quad , \quad \mu_t = n_t\theta_t$$

$$\log(\mu_t) = x_t^T\beta + \log(n_t)$$
$$= \beta_0 + \beta_1 \text{time}_t + \beta_2 \text{ time since rollout}_t + \beta_3 \text{ time post rollout}_t + \beta_4 I\text{ (rollout stage}_t)$$
$$+\beta_5 I\text{ (post rollout stage}_t) + \beta_6 I\text{ (winter}_t) + \beta_7 I\text{ (spring}_t) + \beta_8 I\text{ (summer}_t)$$
$$+ \log\text{ (population estimate}_t)$$

Under this specification, μ_t and ϕ are the location and scale parameters, n_t is the exposure at time t, $\theta_t = \exp(x_t^T\beta)$ and y_t is the count of hospitalisations during discrete time interval t with $t = 1 \ldots T$ being the time values included in the data. The other terms are as follows:

$time_t$ is the discrete time (in months) starting from zero such that the intercept is aligned with July 2004 - the start of the series

$time\ since\ rollout_t$ is the time (in months) since the start of the rollout (starts Jan 2007)

$time\ post\ rollout_t$ is the time (in months) since the end of the rollout stage (starts Jan 2008)

$I(rollout\ stage_t)$ is an indicator variable set to one in the rollout stage and zero otherwise

$I(post\ rollout\ stage_t)$ is an indicator variable set to one in the post rollout stage and zero otherwise

$I(winter_t)$, $I(spring_t)$ and $I(summer_t)$ are the winter, spring, and summer indicator variables (autumn being the reference level)

Holding all other terms constant, the parameters are interpreted (on the log scale) as:

β_0 the overall pre-intervention intercept (in the language of timeseries decomposition, the pre-intervention level [vertical displacement from zero])

β_1 the change in the linear predictor for a month increase in time (the pre-intervention trend)

β_2 the change from the pre-intervention trend (β_1) associated with the rollout phase

β_3 the change from the pre-intervention trend (β_1) associated with the post-rollout phase

β_4 the level change associated with the rollout period

β_5 the level change associated with the post intervention stage

β_6 the change in the linear predictor that was associated with the periodic winter seasonality

β_7 the change in the linear predictor that was associated with the periodic spring seasonality

β_8 the change in the linear predictor that was associated with the periodic summer seasonality

$\log(population\ estimate_t)$ the usual offset as used in count models with variable denominators

The exponentiated parameter estimates give the commonly known rate-ratios, which can be readily translated into a percentage change from the reference level holding all other terms constant. For the series where outbreaks occurred during 2010, we additionally introduce an indicator variable for the outbreak and fitted a term for that covariate in the model.

eMaterials 2. A narrative description of fitting the timeseries for a specific age-class

Example analysis for age class < 12 months

The <12 months year old age group data is used as an example. We applied an analogous approach for the other age-classes.

We fit the specification detailed in Equation 1 to the all-cause acute gastroenteritis (ACG) and rotavirus (RV) coded hospitalisation timeseries for the <12 months cohort and refer to this as Model 1. While the specification is aligned with the standard ITS model suggested in the literature, e.g. [1][3], visual inspection (figure 1) of the ACG and RV-coded hospitalisation series for the <12 months age cohort suggests potential over-specification as no trends are apparent in the pre, nor post-rollout periods. Additionally, the periodicity that appears in the pre-intervention stage all but vanishes in the post-rollout stage. In terms of model specification, this might warrant consideration of an interaction term between stage and seasonality. However, while periodicity is clearly evident in the pre-intervention stage, the peaks occur at different times of the year, albeit typically Winter and Spring. Notwithstanding these possible limitations, the parameter estimates for the ACG and RV-specific series are shown below.

	Model 1 (ACG)					Model 1 (RV specific)				
	Estimate	SE	Lower#	Upper#	p-value	Estimate	SE	Lower#	Upper#	p-value
Intercept	-3.79	0.098	-3.98	-3.60	<0.001***	-6.58	0.359	-7.28	-5.87	<0.001***
Pre-vac trend	0.005	0.005	-0.005	0.015	0.302	0.003	0.018	-0.032	0.038	0.879
Change in pre-vac trend during rollout	-0.023	0.026	-0.074	0.028	0.367	-0.032	0.072	-0.173	0.109	0.653
Change in pre-vac trend post-rollout	-0.009	0.006	-0.021	0.003	0.099	-0.03	0.019	-0.067	0.007	0.118
Change in level during rollout	-0.122	0.19	-0.494	0.25	0.521	0.163	0.583	-0.98	1.31	0.78
Change in level during post-rollout	-0.555	0.177	-0.902	-0.208	0.002**	-0.715	0.579	-1.85	0.42	0.217
Winter	0.138	0.091	-0.04	0.316	0.129	1.19	0.268	0.661	1.71	<0.001***
Spring	0.131	0.091	-0.047	0.309	0.149	1.09	0.295	0.511	1.67	<0.001***
Summer	0.022	0.086	-0.147	0.191	0.798	0.129	0.295	-0.449	0.707	0.662

Scale	18.8	4.10	10.8	26.9	<0.001***	2.93	1.01	0.945	4.91	0.004**
AIC	810.					453.				
#Lower and Upper show 95% CI bounds<br>*** < 0.001, ** < 0.01, * < 0.05										

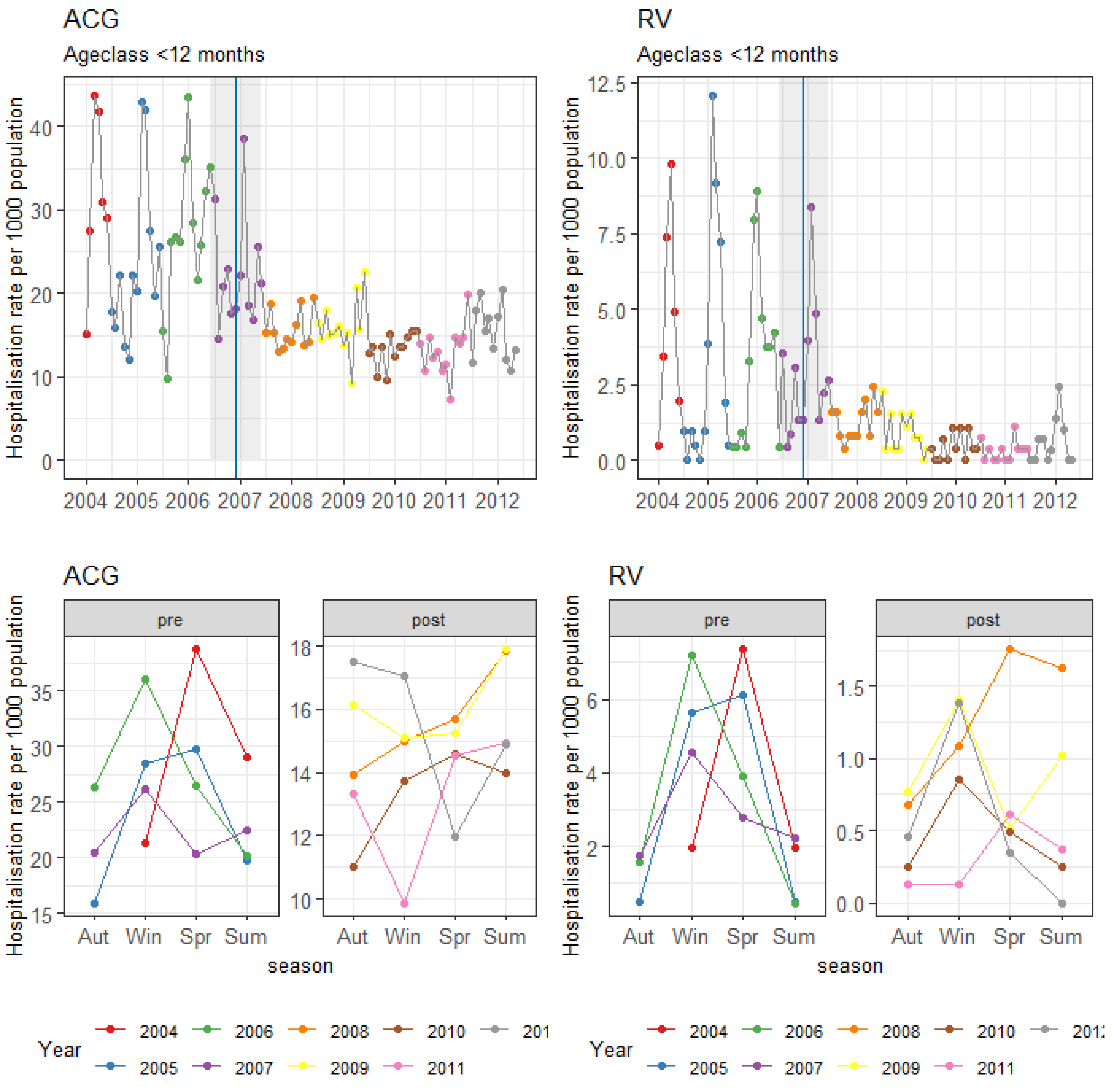

Figure 1 Observed mean monthly hospitalisation rates (and seasonality) per 1000 population for age <12 months for ACG (LHS) and RV (RHS) data

Interpretation of residual diagnostics for the exponential family with non-identity link functions can be unreliable for reasons such as the variance of the data is often expected to change with fitted values, normality is not necessarily going to be apparent and patterns are the norm rather than the exception for plots of predicted versus residuals. Model diagnostics (quantile-quantile plot, residual vs fitted, residual vs leverage and autocorrelation function, figure 2) for Model 1 for the ACG series suggest some deviation from distributional assumptions but certainly not extreme. However, serial dependence is readily detected which would lead to biased standard errors if not accounted for via the NW covariance estimator in the case of a GLM or explicitly modelled as we do here. The pattern in the ACF (and PACF, not shown) suggests a mild autoregressive process of order 1, which was also suggested by a Durbin Watson test (significant at the 0.001 level). Structure was also apparent in the residual plots when plotted against the month and month since vaccine variables (not shown) suggesting another area of potential misspecification.

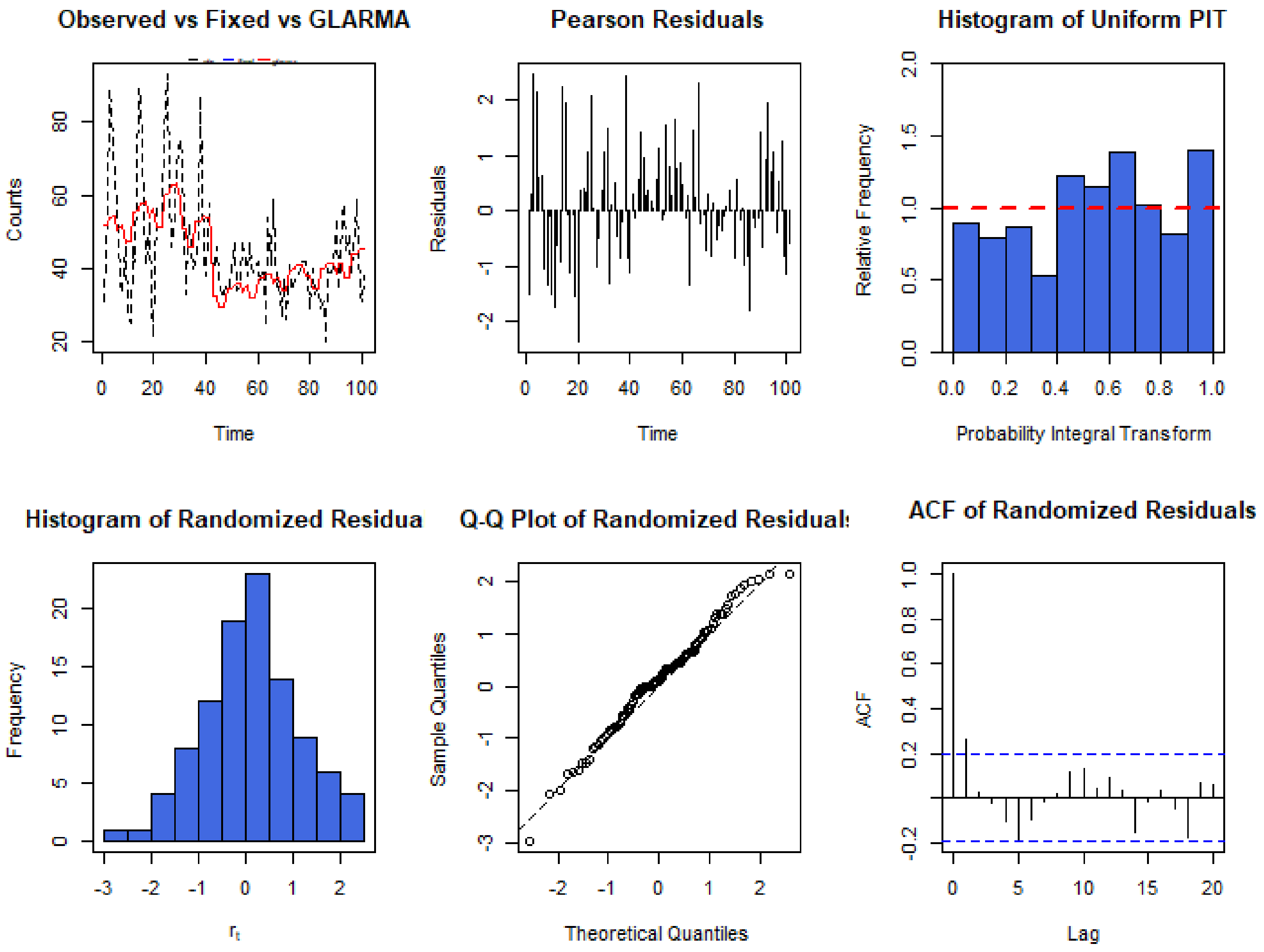

Figure 2 Residual diagnostics for ACG series from Model 1

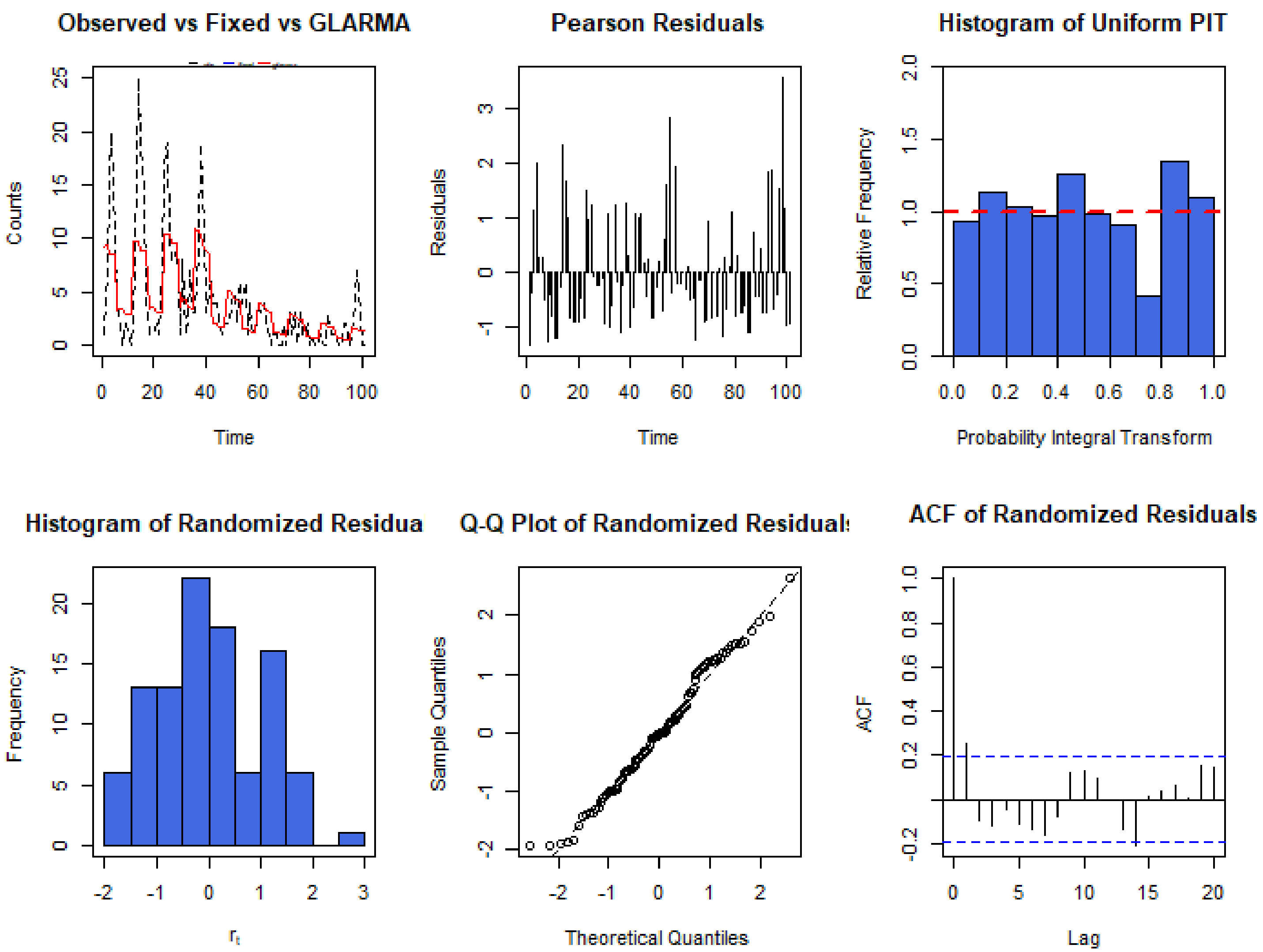

Figure 3 Residual diagnostics for RV series from Model 1

Given the serial dependence in both series, we refit the models retaining the original specification but adding an AR(1) component into the state vector. The parameter estimates from both models are shown below. For both series, Akaike Information Criterion (AIC) and likelihood ratio tests (LRT) suggest that Model 2 has better goodness-of-fit for both the ACG and RV series with LRTs significant at the < 0.01 and < 0.001, respectively.

	Model 2 (ACG)					Model 2 (RV specific)				
	Estimate	SE	Lower#	Upper#	p-value	Estimate	SE	Lower#	Upper#	p-value
Intercept	-3.793	0.115	-4.018	-3.568	<0.001***	-6.809	0.519	-7.826	-5.792	<0.001***
Pre-vac trend	0.006	0.006	-0.006	0.018	0.366	0.024	0.028	-0.031	0.079	0.394
Change in pre-vac trend during rollout	-0.017	0.028	-0.072	0.038	0.557	-0.084	0.099	-0.278	0.11	0.399
Change in pre-vac trend post-rollout	-0.004	0.007	-0.018	0.01	0.565	-0.048	0.031	-0.109	0.013	0.121
Change in level during rollout	-0.106	0.202	-0.502	0.29	0.6	0.424	0.822	-1.187	2.035	0.606
Change in level during post-rollout	-0.783	0.225	-1.224	-0.342	0.001**	-1.335	0.957	-3.211	0.541	0.163
Winter	0.087	0.089	-0.087	0.261	0.33	0.864	0.348	0.182	1.546	0.013*
Spring	0.065	0.097	-0.125	0.255	0.502	0.736	0.376	-0.001	1.473	0.051
Summer	0.015	0.086	-0.154	0.184	0.863	-0.239	0.4	-1.023	0.545	0.549
AR(1)	0.079	0.023	0.034	0.124	0.001**	0.337	0.094	0.153	0.521	<0.001***
Scale	18.308	5.334	7.854	28.762	0.001**	4.795	1.309	2.229	7.361	<0.001***
AIC	804.2					442				
#Lower and Upper show 95% CI bounds										

Model diagnostics (figure 4, 5) were improved for both series with residual serial correlation no longer significant.

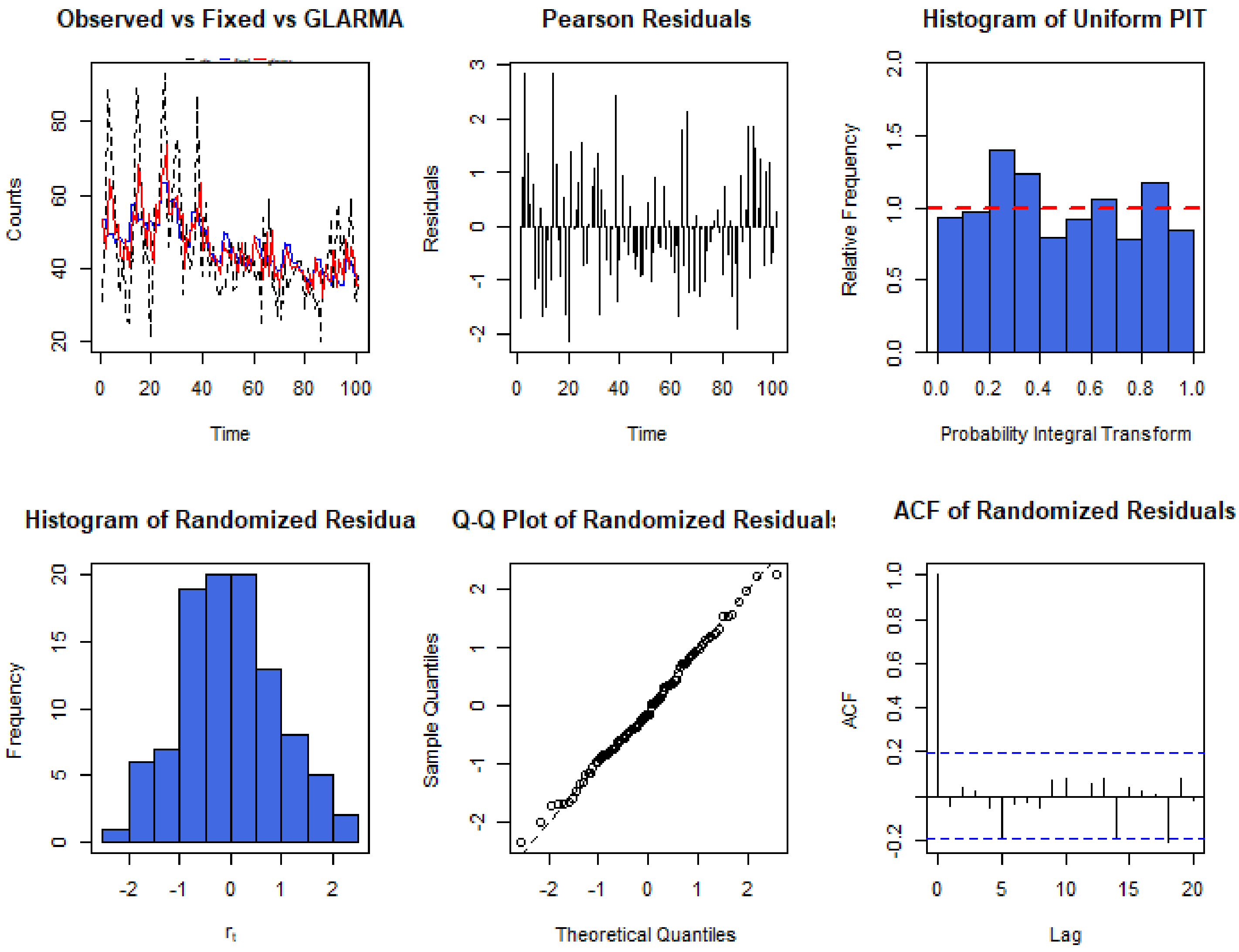

Figure 4 Residual diagnostics for ACG series from Model 2

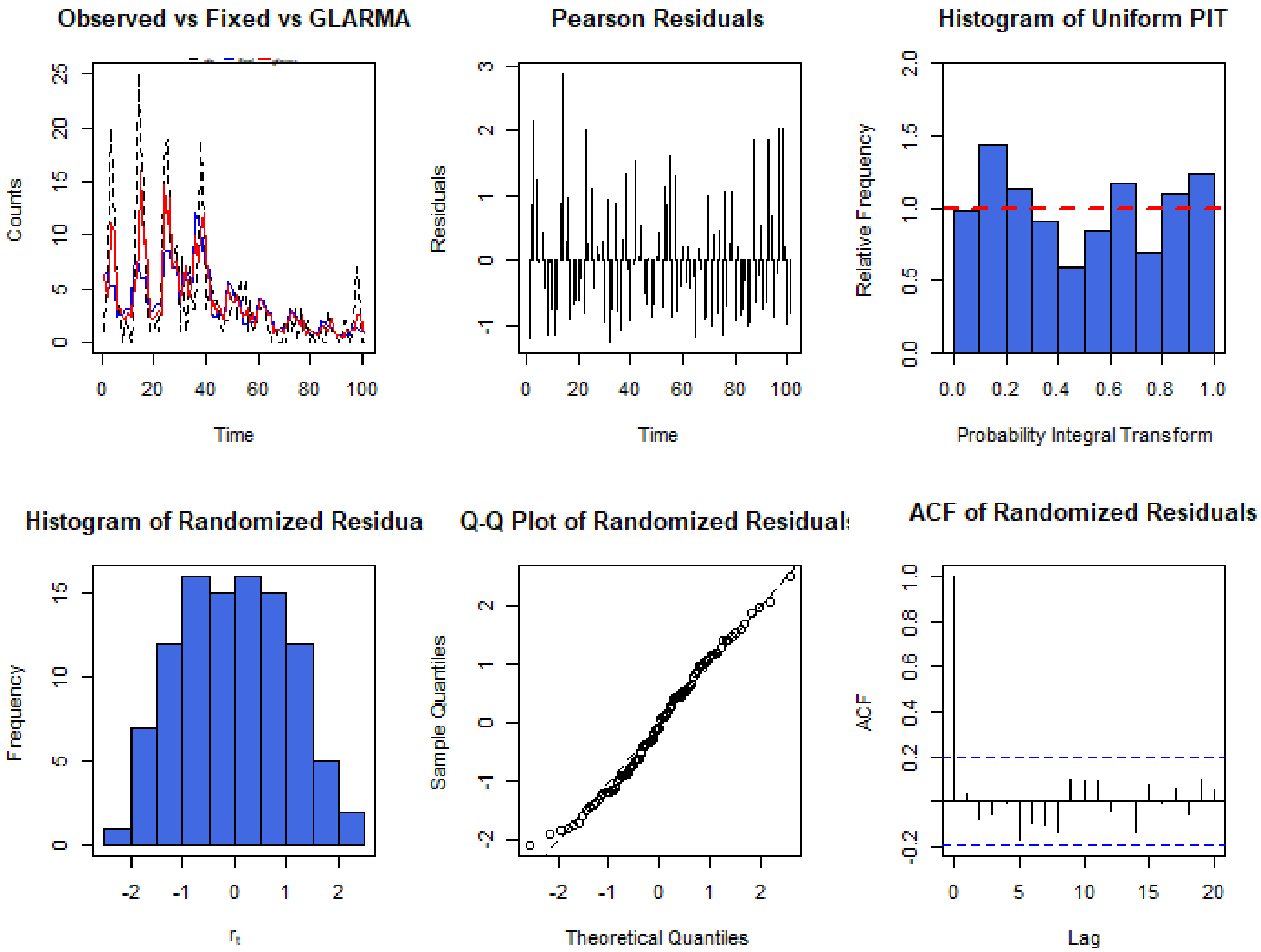

Figure 5 Residual diagnostics for RV series from Model 2

Based on the parameter estimates from Model 2, there is negligible evidence to support a pre-intervention trend, nor changes from the pre-intervention trend in the rollout or post-rollout period in either series. However, Model 2 (ACG) does suggest a -0.783 (95CI -1.224 to -0.342) change in linear predictor when moving from the pre to the post-rollout period, holding all other parameters constant. This corresponds to a 0.46 (95CI 0.29 to 0.71) rate ratio or approximately 54% reduction (on average) in the hospitalisation rate across the whole post-rollout period relative to the pre-intervention period. Model 2 (ACG) has little support for seasonality with an LRT comparing nested models for the ACG series with being non-significant (p-value = 0.7). We think this could relate to what is clear by visual inspection, namely that the periodicity that we see in the pre-intervention period vanishes in the post-rollout period.

For the RV series, Model 2 suggests some support for seasonality, which was confirmed by a significant LRT at the 0.001 level comparing nested models for the RV series with and without a seasonality term. However, we note that AIC was only fractionally improved in the more complex model.

We can get some additional insight into the magnitude of change by comparing the modelled pre-intervention and post-rollout hospitalisation rates by averaging across the fitted values and bootstrapping to estimate the uncertainty. Doing so suggests that the median hospitalisation rate reduced, on average, by 11.78 (95CI 8.89to 14.9) hospitalisations per 1000 population for the ACG series and 2.69 (95CI 1.32 to 5.25) in the RV series. Moreover, given that there are no substantive trends in either series, this difference remains approximately constant over the full post-rollout period.

While it would be possible to remove redundant terms from the models, we retained the full specification as we have assumed that the direct effects associated with the vaccine will follow the pre-specified impact model.

Importantly, we note that a-priori specification does not protect us from the possibility of mis-specification - we do not know the true model for the data generation process. Our primary goal is to examine the strength of evidence for the original model.

eTable 1. Parameter estimates from the GLARMA models fitted to the acute gastroenteritis-coded hospitalisations

age	parameter	description	estimate	se	lower	upper	p-value	ci	rrci
<12 months	beta_0	Intercept	-3.793	0.115	-4.018	-3.568	<0.001	-3.793 (95CI -4.018 to -3.568)	0.02 (95CI 0.02 to 0.03)
<12 months	beta_1	Pre-vac trend	0.006	0.006	-0.006	0.018	0.366	0.006 (95CI -0.006 to 0.018)	1.01 (95CI 0.99 to 1.02)
<12 months	beta_2	Change in pre-vac trend during rollout	-0.017	0.028	-0.072	0.038	0.557	-0.017 (95CI -0.072 to 0.038)	0.98 (95CI 0.93 to 1.04)
<12 months	beta_3	Change in pre-vac trend post-rollout	-0.004	0.007	-0.018	0.01	0.565	-0.004 (95CI -0.018 to 0.01)	1 (95CI 0.98 to 1.01)
<12 months	beta_4	Change in level during rollout	-0.106	0.202	-0.502	0.29	0.6	-0.106 (95CI -0.502 to 0.29)	0.9 (95CI 0.61 to 1.34)
<12 months	beta_5	Change in level during post-rollout	-0.783	0.225	-1.224	-0.342	0.001	-0.783 (95CI -1.224 to -0.342)	0.46 (95CI 0.29 to 0.71)
<12 months	beta_6	Winter	0.087	0.089	-0.087	0.261	0.33	0.087 (95CI -0.087 to 0.261)	1.09 (95CI 0.92 to 1.3)
<12 months	beta_7	Spring	0.065	0.097	-0.125	0.255	0.502	0.065 (95CI -0.125 to 0.255)	1.07 (95CI 0.88 to 1.29)
<12 months	beta_8	Summer	0.015	0.086	-0.154	0.184	0.863	0.015 (95CI -0.154 to 0.184)	1.02 (95CI 0.86 to 1.2)
<12 months	phi_1	AR(1)	0.079	0.023	0.034	0.124	0.001	0.079 (95CI 0.034 to 0.124)	
<12 months	sigma	Scale	18.308	5.334	7.854	28.762	0.001	18.308 (95CI 7.854 to 28.762)	
<12 months	AIC	AIC	804.2						
12-23 months	beta_0	Intercept	-3.761	0.148	-4.051	-3.471	<0.001	-3.761 (95CI -4.051 to -3.471)	0.02 (95CI 0.02 to 0.03)
12-23 months	beta_1	Pre-vac trend	-0.002	0.008	-0.018	0.014	0.754	-0.002 (95CI -0.018 to 0.014)	1 (95CI 0.98 to 1.01)

12-23 months	beta_2	Change in pre-vac trend during rollout	0.091	0.069	-0.044	0.226	0.185	0.091 (95CI -0.044 to 0.226)	1.1 (95CI 0.96 to 1.25)
12-23 months	beta_3	Change in pre-vac trend post-rollout	-0.004	0.009	-0.022	0.014	0.626	-0.004 (95CI -0.022 to 0.014)	1 (95CI 0.98 to 1.01)
12-23 months	beta_4	Change in level during rollout	-0.717	0.541	-1.777	0.343	0.185	-0.717 (95CI -1.777 to 0.343)	0.49 (95CI 0.17 to 1.41)
12-23 months	beta_5	Change in level during post-rollout	-0.448	0.276	-0.989	0.093	0.104	-0.448 (95CI -0.989 to 0.093)	0.64 (95CI 0.37 to 1.1)
12-23 months	beta_6	Winter	0.195	0.092	0.015	0.375	0.034	0.195 (95CI 0.015 to 0.375)	1.22 (95CI 1.02 to 1.45)
12-23 months	beta_7	Spring	0.198	0.099	0.004	0.392	0.045	0.198 (95CI 0.004 to 0.392)	1.22 (95CI 1 to 1.48)
12-23 months	beta_8	Summer	0.089	0.088	-0.083	0.261	0.309	0.089 (95CI -0.083 to 0.261)	1.09 (95CI 0.92 to 1.3)
12-23 months	phi_1	AR(1)	0.156	0.028	0.101	0.211	<0.001	0.156 (95CI 0.101 to 0.211)	
12-23 months	sigma	Scale	23.526	7.097	9.616	37.436	0.001	23.526 (95CI 9.616 to 37.436)	
12-23 months	AIC	AIC	769.3						
2 years	beta_0	Intercept	-4.527	0.149	-4.819	-4.235	<0.001	-4.527 (95CI -4.819 to -4.235)	0.01 (95CI 0.01 to 0.01)
2 years	beta_1	Pre-vac trend	0.002	0.008	-0.014	0.018	0.821	0.002 (95CI -0.014 to 0.018)	1 (95CI 0.99 to 1.02)
2 years	beta_2	Change in pre-vac trend during rollout	-0.079	0.045	-0.167	0.009	0.082	-0.079 (95CI -0.167 to 0.009)	0.92 (95CI 0.85 to 1.01)
2 years	beta_3	Change in pre-vac trend post-rollout	-0.005	0.01	-0.025	0.015	0.577	-0.005 (95CI -0.025 to 0.015)	1 (95CI 0.98 to 1.02)
2 years	beta_4	Change in level during rollout	0.313	0.37	-0.412	1.038	0.397	0.313 (95CI -0.412 to 1.038)	1.37 (95CI 0.66 to 2.82)
2 years	beta_5	Change in level during post-rollout	-0.557	0.27	-1.086	-0.028	0.039	-0.557 (95CI -1.086 to -0.028)	0.57 (95CI 0.34 to 0.97)
2 years	beta_6	Winter	0.253	0.089	0.079	0.427	0.004	0.253 (95CI 0.079 to 0.427)	1.29 (95CI 1.08 to 1.53)

2 years	beta_7	Spring	0.181	0.107	-0.029	0.391	0.089	0.181 (95CI -0.029 to 0.391)	1.2 (95CI 0.97 to 1.48)
2 years	beta_8	Summer	-0.057	0.097	-0.247	0.133	0.555	-0.057 (95CI -0.247 to 0.133)	0.94 (95CI 0.78 to 1.14)
2 years	phi_1	AR(1)	0.174	0.029	0.117	0.231	<0.001	0.174 (95CI 0.117 to 0.231)	
2 years	sigma	Scale	17.271	15.339	-12.793	47.335	0.26	17.271 (95CI -12.793 to 47.335)	
2 years	AIC	AIC	663.5						
3 years	beta_0	Intercept	-4.98	0.156	-5.286	-4.674	<0.001	-4.98 (95CI -5.286 to -4.674)	0.01 (95CI 0.01 to 0.01)
3 years	beta_1	Pre-vac trend	-0.006	0.008	-0.022	0.01	0.475	-0.006 (95CI -0.022 to 0.01)	0.99 (95CI 0.98 to 1.01)
3 years	beta_2	Change in pre-vac trend during rollout	0.019	0.041	-0.061	0.099	0.639	0.019 (95CI -0.061 to 0.099)	1.02 (95CI 0.94 to 1.1)
3 years	beta_3	Change in pre-vac trend post-rollout	0.005	0.009	-0.013	0.023	0.587	0.005 (95CI -0.013 to 0.023)	1.01 (95CI 0.99 to 1.02)
3 years	beta_4	Change in level during rollout	0.111	0.285	-0.448	0.67	0.697	0.111 (95CI -0.448 to 0.67)	1.12 (95CI 0.64 to 1.95)
3 years	beta_5	Change in level during post-rollout	-0.299	0.297	-0.881	0.283	0.314	-0.299 (95CI -0.881 to 0.283)	0.74 (95CI 0.41 to 1.33)
3 years	beta_6	Winter	0.248	0.117	0.019	0.477	0.034	0.248 (95CI 0.019 to 0.477)	1.28 (95CI 1.02 to 1.61)
3 years	beta_7	Spring	0.165	0.131	-0.092	0.422	0.208	0.165 (95CI -0.092 to 0.422)	1.18 (95CI 0.91 to 1.53)
3 years	beta_8	Summer	-0.195	0.141	-0.471	0.081	0.166	-0.195 (95CI -0.471 to 0.081)	0.82 (95CI 0.62 to 1.08)
3 years	beta_9	Outbreak	0.598	0.123	0.357	0.839	<0.001	0.598 (95CI 0.357 to 0.839)	1.82 (95CI 1.43 to 2.31)
3 years	phi_1	AR(1)	0.126	0.033	0.061	0.191	<0.001	0.126 (95CI 0.061 to 0.191)	
3 years	sigma	Scale	7.015	9.232	-11.079	25.109	0.447	7.015 (95CI -11.079 to 25.109)	
3 years	AIC	AIC	627.3						
4 years	beta_0	Intercept	-5.316	0.283	-5.871	-4.761	<0.001	-5.316 (95CI -5.871 to -4.761)	0 (95CI 0 to 0.01)

4 years	beta_1	Pre-vac trend	0.009	0.015	-0.02	0.038	0.53	0.009<br>(95CI -0.02 to 0.038)	1.01<br>(95CI 0.98 to 1.04)
4 years	beta_2	Change in pre-vac trend during rollout	-0.034	0.081	-0.193	0.125	0.679	-0.034<br>(95CI -0.193 to 0.125)	0.97<br>(95CI 0.82 to 1.13)
4 years	beta_3	Change in pre-vac trend post-rollout	-0.007	0.016	-0.038	0.024	0.646	-0.007<br>(95CI -0.038 to 0.024)	0.99<br>(95CI 0.96 to 1.02)
4 years	beta_4	Change in level during rollout	-0.264	0.715	-1.665	1.137	0.712	-0.264<br>(95CI -1.665 to 1.137)	0.77<br>(95CI 0.19 to 3.12)
4 years	beta_5	Change in level during post-rollout	-0.956	0.5	-1.936	0.024	0.056	-0.956<br>(95CI -1.936 to 0.024)	0.38<br>(95CI 0.14 to 1.02)
4 years	beta_6	Winter	-0.097	0.203	-0.495	0.301	0.634	-0.097<br>(95CI -0.495 to 0.301)	0.91<br>(95CI 0.61 to 1.35)
4 years	beta_7	Spring	0.083	0.218	-0.344	0.51	0.702	0.083<br>(95CI -0.344 to 0.51)	1.09<br>(95CI 0.71 to 1.67)
4 years	beta_8	Summer	-0.187	0.238	-0.653	0.279	0.432	-0.187<br>(95CI -0.653 to 0.279)	0.83<br>(95CI 0.52 to 1.32)
4 years	beta_9	Outbreak	0.76	0.223	0.323	1.197	0.001	0.76<br>(95CI 0.323 to 1.197)	2.14<br>(95CI 1.38 to 3.31)
4 years	phi_1	AR(1)	0.093	0.076	-0.056	0.242	0.225	0.093<br>(95CI -0.056 to 0.242)	
4 years	sigma	Scale	19.904	4.215	11.643	28.165	<0.001	19.904<br>(95CI 11.643 to 28.165)	
4 years	AIC	AIC	575						
5-9 years	beta_0	Intercept	-6.04	0.174	-6.381	-5.699	<0.001	-6.04<br>(95CI -6.381 to -5.699)	0<br>(95CI 0 to 0)
5-9 years	beta_1	Pre-vac trend	0	0.009	-0.018	0.018	0.978	0<br>(95CI -0.018 to 0.018)	1<br>(95CI 0.98 to 1.02)
5-9 years	beta_2	Change in pre-vac trend during rollout	0.041	0.044	-0.045	0.127	0.355	0.041<br>(95CI -0.045 to 0.127)	1.04<br>(95CI 0.96 to 1.14)
5-9 years	beta_3	Change in pre-vac trend post-rollout	0.007	0.009	-0.011	0.025	0.475	0.007<br>(95CI -0.011 to 0.025)	1.01<br>(95CI 0.99 to 1.03)
5-9 years	beta_4	Change in level during rollout	-0.193	0.367	-0.912	0.526	0.599	-0.193<br>(95CI -0.912 to 0.526)	0.82<br>(95CI 0.4 to 1.69)

5-9 years	beta_5	Change in level during post-rollout	-0.484	0.267	-1.007	0.039	0.07	-0.484 (95CI -1.007 to 0.039)	0.62 (95CI 0.37 to 1.04)
5-9 years	beta_6	Winter	0.069	0.113	-0.152	0.29	0.545	0.069 (95CI -0.152 to 0.29)	1.07 (95CI 0.86 to 1.34)
5-9 years	beta_7	Spring	0.147	0.101	-0.051	0.345	0.147	0.147 (95CI -0.051 to 0.345)	1.16 (95CI 0.95 to 1.41)
5-9 years	beta_8	Summer	0.079	0.103	-0.123	0.281	0.441	0.079 (95CI -0.123 to 0.281)	1.08 (95CI 0.88 to 1.32)
5-9 years	beta_9	Outbreak	0.206	0.105	0	0.412	0.05	0.206 (95CI 0 to 0.412)	1.23 (95CI 1 to 1.51)
5-9 years	phi_1	AR(1)	0.1	0.027	0.047	0.153	<0.001	0.1 (95CI 0.047 to 0.153)	
5-9 years	sigma	Scale	38.818	15.448	8.54	69.096	0.012	38.818 (95CI 8.54 to 69.096)	
5-9 years	AIC	AIC	684.4						
10-19 years	beta_0	Intercept	-6.427	0.089	-6.601	-6.253	<0.001	-6.427 (95CI -6.601 to -6.253)	0 (95CI 0 to 0)
10-19 years	beta_1	Pre-vac trend	0.004	0.004	-0.004	0.012	0.33	0.004 (95CI -0.004 to 0.012)	1 (95CI 1 to 1.01)
10-19 years	beta_2	Change in pre-vac trend during rollout	0.027	0.015	-0.002	0.056	0.071	0.027 (95CI -0.002 to 0.056)	1.03 (95CI 1 to 1.06)
10-19 years	beta_3	Change in pre-vac trend post-rollout	0.002	0.005	-0.008	0.012	0.606	0.002 (95CI -0.008 to 0.012)	1 (95CI 0.99 to 1.01)
10-19 years	beta_4	Change in level during rollout	-0.231	0.138	-0.501	0.039	0.095	-0.231 (95CI -0.501 to 0.039)	0.79 (95CI 0.61 to 1.04)
10-19 years	beta_5	Change in level during post-rollout	-0.339	0.127	-0.588	-0.09	0.008	-0.339 (95CI -0.588 to -0.09)	0.71 (95CI 0.56 to 0.91)
10-19 years	beta_6	Winter	-0.03	0.056	-0.14	0.08	0.596	-0.03 (95CI -0.14 to 0.08)	0.97 (95CI 0.87 to 1.08)
10-19 years	beta_7	Spring	0.054	0.059	-0.062	0.17	0.356	0.054 (95CI -0.062 to 0.17)	1.06 (95CI 0.94 to 1.19)
10-19 years	beta_8	Summer	0.097	0.058	-0.017	0.211	0.098	0.097 (95CI -0.017 to 0.211)	1.1 (95CI 0.98 to 1.23)

10-19 years	sigma	Scale	59.855	60.478	-58.68	178.39	0.322	59.855 (95CI -58.68 to 178.39)	
10-19 years	AIC	AIC	726.9						
20-44 years	beta_0	Intercept	-5.574	0.037	-5.647	-5.501	<0.001	-5.574 (95CI -5.647 to -5.501)	0 (95CI 0 to 0)
20-44 years	beta_1	Pre-vac trend	0.002	0.002	-0.002	0.006	0.3	0.002 (95CI -0.002 to 0.006)	1 (95CI 1 to 1.01)
20-44 years	beta_2	Change in pre-vac trend during rollout	0.006	0.012	-0.018	0.03	0.599	0.006 (95CI -0.018 to 0.03)	1.01 (95CI 0.98 to 1.03)
20-44 years	beta_3	Change in pre-vac trend post-rollout	0.002	0.002	-0.002	0.006	0.277	0.002 (95CI -0.002 to 0.006)	1 (95CI 1 to 1.01)
20-44 years	beta_4	Change in level during rollout	-0.003	0.07	-0.14	0.134	0.962	-0.003 (95CI -0.14 to 0.134)	1 (95CI 0.87 to 1.14)
20-44 years	beta_5	Change in level during post-rollout	-0.134	0.069	-0.269	0.001	0.052	-0.134 (95CI -0.269 to 0.001)	0.87 (95CI 0.76 to 1)
20-44 years	beta_6	Winter	-0.092	0.032	-0.155	-0.029	0.004	-0.092 (95CI -0.155 to -0.029)	0.91 (95CI 0.86 to 0.97)
20-44 years	beta_7	Spring	0.005	0.027	-0.048	0.058	0.842	0.005 (95CI -0.048 to 0.058)	1.01 (95CI 0.95 to 1.06)
20-44 years	beta_8	Summer	-0.006	0.026	-0.057	0.045	0.822	-0.006 (95CI -0.057 to 0.045)	0.99 (95CI 0.94 to 1.05)
20-44 years	sigma	Scale	180.782	42.862	96.774	264.79	<0.001	180.782 (95CI 96.774 to 264.79)	
20-44 years	AIC	AIC	959.6						
45-64 years	beta_0	Intercept	-5.381	0.049	-5.477	-5.285	<0.001	-5.381 (95CI -5.477 to -5.285)	0 (95CI 0 to 0.01)
45-64 years	beta_1	Pre-vac trend	0.008	0.003	0.002	0.014	0.003	0.008 (95CI 0.002 to 0.014)	1.01 (95CI 1 to 1.01)
45-64 years	beta_2	Change in pre-vac trend during rollout	-0.007	0.01	-0.027	0.013	0.497	-0.007 (95CI -0.027 to 0.013)	0.99 (95CI 0.97 to 1.01)
45-64 years	beta_3	Change in pre-vac trend post-rollout	-0.006	0.003	-0.012	0	0.054	-0.006 (95CI -0.012 to 0)	0.99 (95CI 0.99 to 1)

45-64 years	beta_4	Change in level during rollout	-0.065	0.081	-0.224	0.094	0.423	-0.065 (95CI -0.224 to 0.094)	0.94 (95CI 0.8 to 1.1)
45-64 years	beta_5	Change in level during post-rollout	-0.104	0.088	-0.276	0.068	0.239	-0.104 (95CI -0.276 to 0.068)	0.9 (95CI 0.76 to 1.07)
45-64 years	beta_6	Winter	-0.023	0.025	-0.072	0.026	0.354	-0.023 (95CI -0.072 to 0.026)	0.98 (95CI 0.93 to 1.03)
45-64 years	beta_7	Spring	0.006	0.03	-0.053	0.065	0.837	0.006 (95CI -0.053 to 0.065)	1.01 (95CI 0.95 to 1.07)
45-64 years	beta_8	Summer	-0.048	0.024	-0.095	-0.001	0.043	-0.048 (95CI -0.095 to -0.001)	0.95 (95CI 0.91 to 1)
45-64 years	phi_3	AR(3)	0.033	0.013	0.008	0.058	0.012	0.033 (95CI 0.008 to 0.058)	
45-64 years	sigma	Scale	356.526	93.333	173.597	539.455	<0.001	356.526 (95CI 173.597 to 539.455)	
45-64 years	AIC	AIC	929.2						
≥65 years	beta_0	Intercept	-4.116	0.044	-4.202	-4.03	<0.001	-4.116 (95CI -4.202 to -4.03)	0.02 (95CI 0.01 to 0.02)
≥65 years	beta_1	Pre-vac trend	-0.001	0.003	-0.007	0.005	0.789	-0.001 (95CI -0.007 to 0.005)	1 (95CI 0.99 to 1.01)
≥65 years	beta_2	Change in pre-vac trend during rollout	0.004	0.013	-0.021	0.029	0.76	0.004 (95CI -0.021 to 0.029)	1 (95CI 0.98 to 1.03)
≥65 years	beta_3	Change in pre-vac trend post-rollout	0.004	0.003	-0.002	0.01	0.188	0.004 (95CI -0.002 to 0.01)	1 (95CI 1 to 1.01)
≥65 years	beta_4	Change in level during rollout	0.165	0.098	-0.027	0.357	0.092	0.165 (95CI -0.027 to 0.357)	1.18 (95CI 0.97 to 1.43)
≥65 years	beta_5	Change in level during post-rollout	0.071	0.098	-0.121	0.263	0.466	0.071 (95CI -0.121 to 0.263)	1.07 (95CI 0.89 to 1.3)
≥65 years	beta_6	Winter	-0.02	0.03	-0.079	0.039	0.504	-0.02 (95CI -0.079 to 0.039)	0.98 (95CI 0.92 to 1.04)
≥65 years	beta_7	Spring	0.016	0.033	-0.049	0.081	0.627	0.016 (95CI -0.049 to 0.081)	1.02 (95CI 0.95 to 1.08)
≥65 years	beta_8	Summer	-0.035	0.031	-0.096	0.026	0.26	-0.035 (95CI -0.096 to 0.026)	0.97 (95CI 0.91 to 1.03)

≥65 years	phi_1	AR(1)	0.037	0.009	0.019	0.055	<0.001	0.037 (95CI 0.019 to 0.055)	
≥65 years	sigma	Scale	158.859	43.059	74.465	243.253	<0.001	158.859 (95CI 74.465 to 243.253)	
≥65 years	AIC	AIC	1028.8						

eTable 2. Parameter estimates from the GLARMA models fitted to the rotavirus-coded hospitalisations

age	parameter	description	estimate	se	lower	upper	p-value	Ci	rrci
<12 months	beta_0	Intercept	-6.809	0.519	-7.826	-5.792	<0.001	-6.809 (95CI -7.826 to -5.792)	0 (95CI 0 to 0)
<12 months	beta_1	Pre-vac trend	0.024	0.028	-0.031	0.079	0.394	0.024 (95CI -0.031 to 0.079)	1.02 (95CI 0.97 to 1.08)
<12 months	beta_2	Change in pre-vac trend during rollout	-0.084	0.099	-0.278	0.11	0.399	-0.084 (95CI -0.278 to 0.11)	0.92 (95CI 0.76 to 1.12)
<12 months	beta_3	Change in pre-vac trend post-rollout	-0.048	0.031	-0.109	0.013	0.121	-0.048 (95CI -0.109 to 0.013)	0.95 (95CI 0.9 to 1.01)
<12 months	beta_4	Change in level during rollout	0.424	0.822	-1.187	2.035	0.606	0.424 (95CI -1.187 to 2.035)	1.53 (95CI 0.31 to 7.65)
<12 months	beta_5	Change in level during post-rollout	-1.335	0.957	-3.211	0.541	0.163	-1.335 (95CI -3.211 to 0.541)	0.26 (95CI 0.04 to 1.72)
<12 months	beta_6	Winter	0.864	0.348	0.182	1.546	0.013	0.864 (95CI 0.182 to 1.546)	2.37 (95CI 1.2 to 4.69)
<12 months	beta_7	Spring	0.736	0.376	-0.001	1.473	0.051	0.736 (95CI -0.001 to 1.473)	2.09 (95CI 1 to 4.36)
<12 months	beta_8	Summer	-0.239	0.4	-1.023	0.545	0.549	-0.239 (95CI -1.023 to 0.545)	0.79 (95CI 0.36 to 1.72)
<12 months	phi_1	AR(1)	0.337	0.094	0.153	0.521	<0.001	0.337 (95CI 0.153 to 0.521)	
<12 months	sigma	Scale	4.795	1.309	2.229	7.361	<0.001	4.795 (95CI 2.229 to 7.361)	
<12 months	AIC	AIC	443.5						
12-23 months	beta_0	Intercept	-6.394	0.504	-7.382	-5.406	<0.001	-6.394 (95CI -7.382 to -5.406)	0 (95CI 0 to 0)
12-23 months	beta_1	Pre-vac trend	-0.004	0.027	-0.057	0.049	0.884	-0.004 (95CI -0.057 to 0.049)	1 (95CI 0.94 to 1.05)

12-23 months	beta_2	Change in pre-vac trend during rollout	-0.074	0.101	-0.272	0.124	0.462	-0.074 (95CI -0.272 to 0.124)	0.93 (95CI 0.76 to 1.13)
12-23 months	beta_3	Change in pre-vac trend post-rollout	-0.021	0.031	-0.082	0.04	0.487	-0.021 (95CI -0.082 to 0.04)	0.98 (95CI 0.92 to 1.04)
12-23 months	beta_4	Change in level during rollout	0.609	0.889	-1.133	2.351	0.494	0.609 (95CI -1.133 to 2.351)	1.84 (95CI 0.32 to 10.5)
12-23 months	beta_5	Change in level during post-rollout	-1.169	0.854	-2.843	0.505	0.171	-1.169 (95CI -2.843 to 0.505)	0.31 (95CI 0.06 to 1.66)
12-23 months	beta_6	Winter	1.049	0.323	0.416	1.682	0.001	1.049 (95CI 0.416 to 1.682)	2.85 (95CI 1.52 to 5.38)
12-23 months	beta_7	Spring	1.074	0.373	0.343	1.805	0.004	1.074 (95CI 0.343 to 1.805)	2.93 (95CI 1.41 to 6.08)
12-23 months	beta_8	Summer	-0.198	0.451	-1.082	0.686	0.66	-0.198 (95CI -1.082 to 0.686)	0.82 (95CI 0.34 to 1.99)
12-23 months	phi_1	AR(1)	0.237	0.092	0.057	0.417	0.01	0.237 (95CI 0.057 to 0.417)	
12-23 months	sigma	Scale	3.598	1.213	1.221	5.975	0.003	3.598 (95CI 1.221 to 5.975)	
12-23 months	AIC	AIC	405.3						
2 years	beta_0	Intercept	-7.398	0.436	-8.253	-6.543	<0.001	-7.398 (95CI -8.253 to -6.543)	0 (95CI 0 to 0)
2 years	beta_1	Pre-vac trend	-0.005	0.017	-0.038	0.028	0.764	-0.005 (95CI -0.038 to 0.028)	1 (95CI 0.96 to 1.03)
2 years	beta_2	Change in pre-vac trend during rollout	-0.131	0.107	-0.341	0.079	0.224	-0.131 (95CI -0.341 to 0.079)	0.88 (95CI 0.71 to 1.08)
2 years	beta_3	Change in pre-vac trend post-rollout	-0.043	0.022	-0.086	0	0.046	-0.043 (95CI -0.086 to 0)	0.96 (95CI 0.92 to 1)
2 years	beta_4	Change in level during rollout	0.53	0.74	-0.92	1.98	0.474	0.53 (95CI -0.92 to 1.98)	1.7 (95CI 0.4 to 7.24)
2 years	beta_5	Change in level during post-rollout	0.003	0.597	-1.167	1.173	0.996	0.003 (95CI -1.167 to 1.173)	1 (95CI 0.31 to 3.23)
2 years	beta_6	Winter	1.186	0.343	0.514	1.858	0.001	1.186 (95CI 0.514 to 1.858)	3.27 (95CI 1.67 to 6.41)

2 years	beta_7	Spring	1.235	0.371	0.508	1.962	0.001	1.235 (95CI 0.508 to 1.962)	3.44 (95CI 1.66 to 7.11)
2 years	beta_8	Summer	0.065	0.387	-0.694	0.824	0.867	0.065 (95CI -0.694 to 0.824)	1.07 (95CI 0.5 to 2.28)
2 years	phi_1	AR(1)	0.24	0.104	0.036	0.444	0.022	0.24 (95CI 0.036 to 0.444)	
2 years	sigma	Scale	1.301	1.467	-1.574	4.176	0.375	1.301 (95CI -1.574 to 4.176)	
2 years	AIC	AIC	330.6						

eMaterials 3. Timeseries and seasonality plots for all the age-classes >12 months

Age class - 12-23 months

The series are structurally similar to the first age class with level, possible seasonality, and noise:

- Possible trends in post-rollout stage
- Change in level when considering pre versus post
- Change in variability
- Periodicity in the pre-intervention period, not as strongly apparent in the post-rollout

Also:

- Increasing number of zeros in the RV series
- Lower rate in RV series

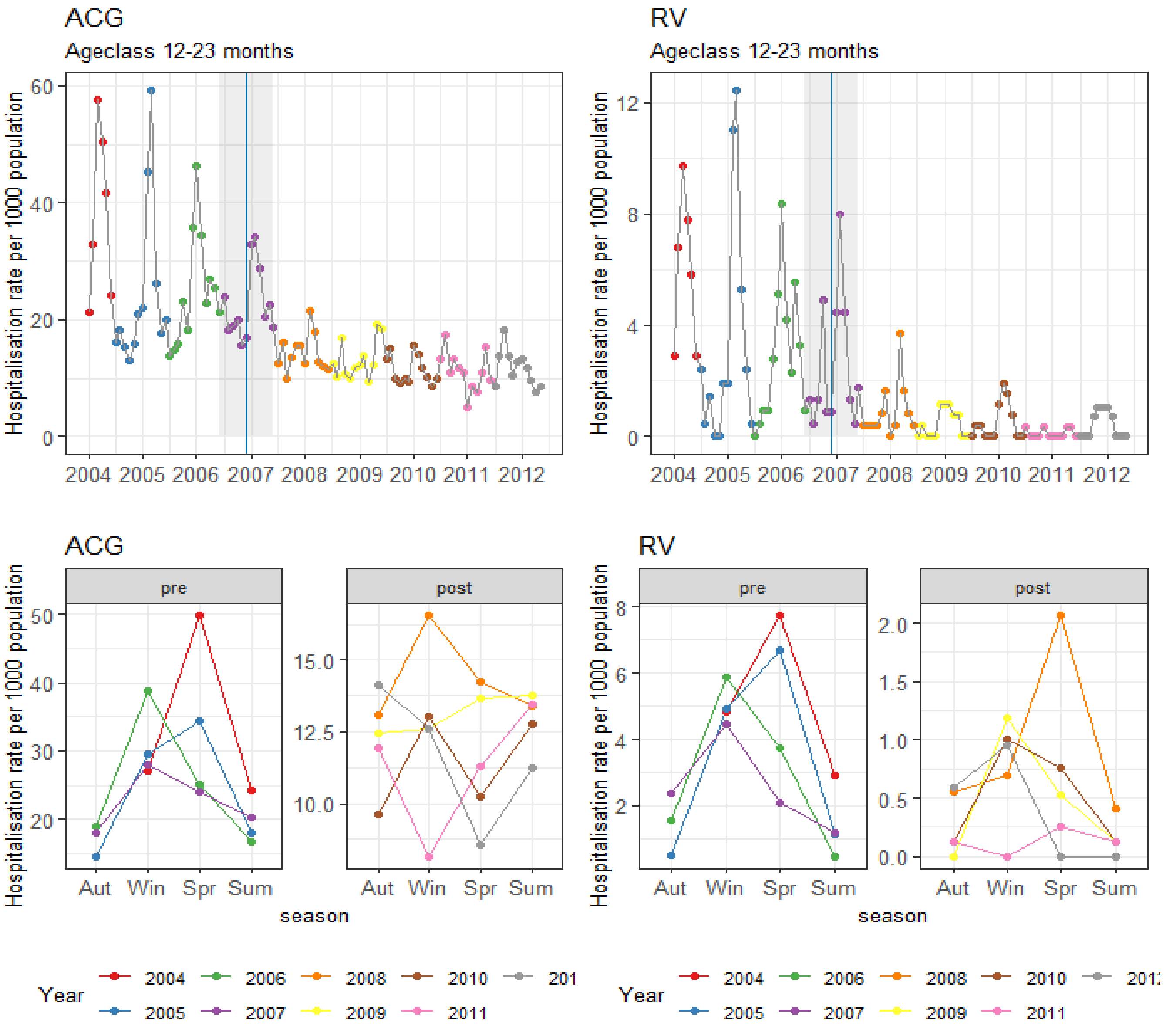

Age class – 2 years (24-35 months)

The series are structurally similar to the first age class with level, possible seasonality, and noise:

- Negligible differential stage trends apparent (clear overall trend)
- Clear change in level when considering pre versus post
- Clear change in variability
- Periodicity in the pre-intervention period, not strongly apparent in the post-rollout

Also:

- Number of zeros in the RV series
- Lower rate in RV series

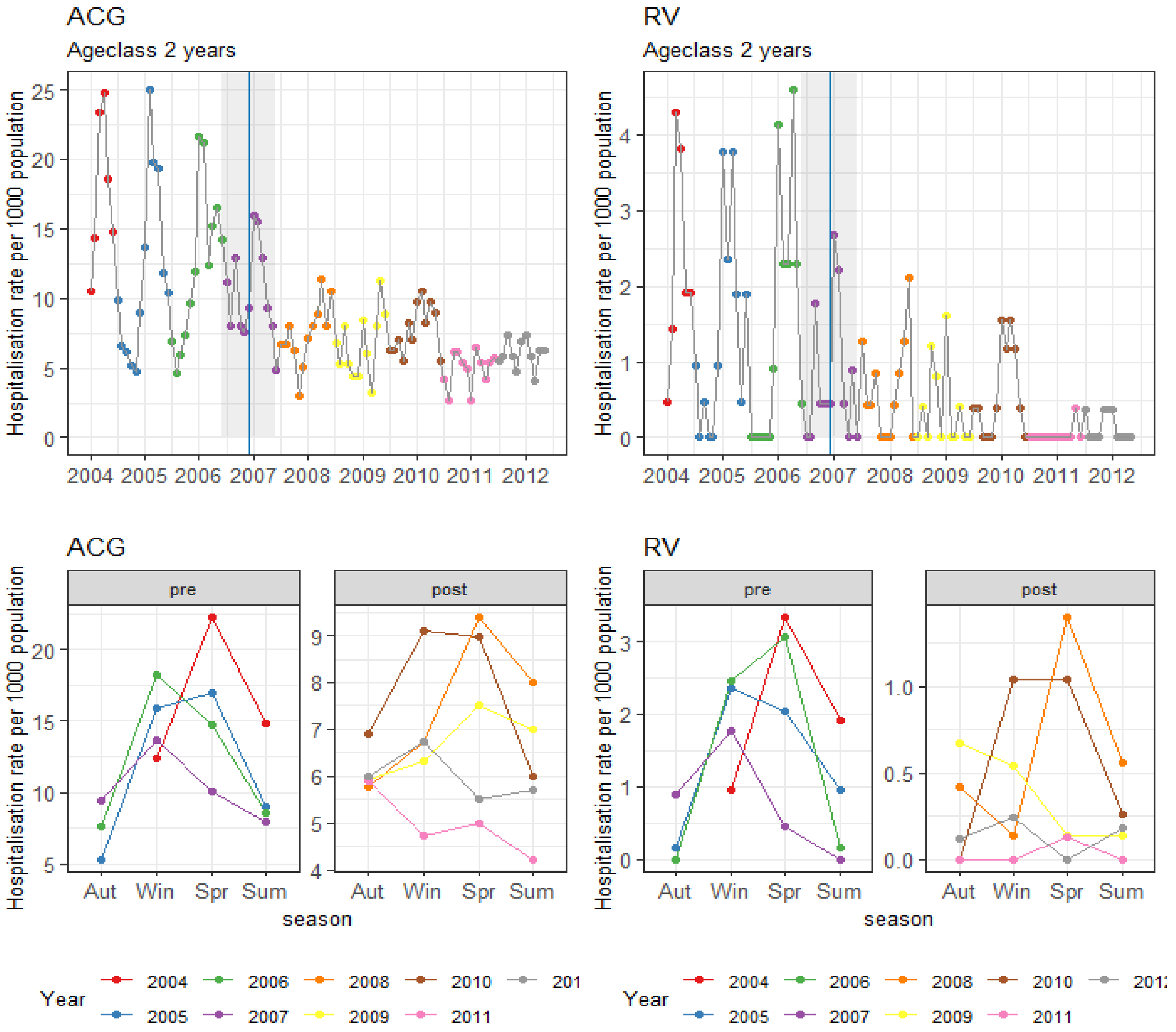

Age class - 3 Years (36-47 months)

The series are structurally similar to the first age class with level, possible seasonality, and noise:

- Negligible differential stage trends apparent (slight overall trend)
- Clear change in level
- Clear change in variability
- Periodicity in the pre-intervention period, not as apparent in the post-rollout

New points of note:

- Larger number of zeros in the RV series
- Peak around 2010 in ACG and RV series
- Lower rate in RV series
- Insufficient data to model RV

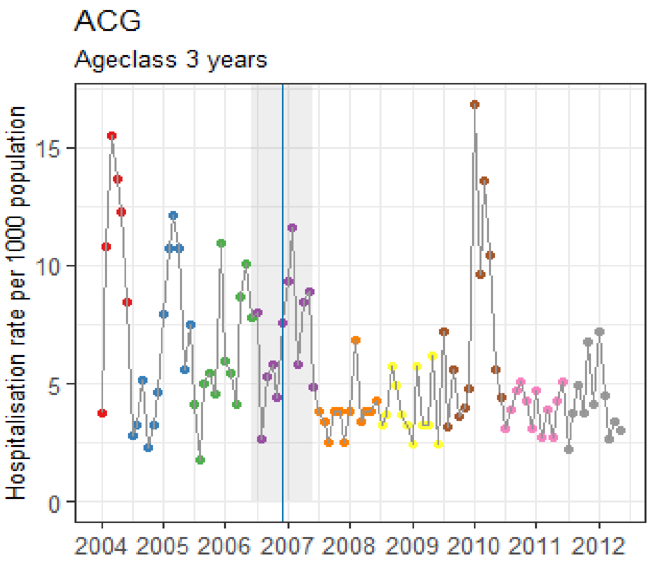

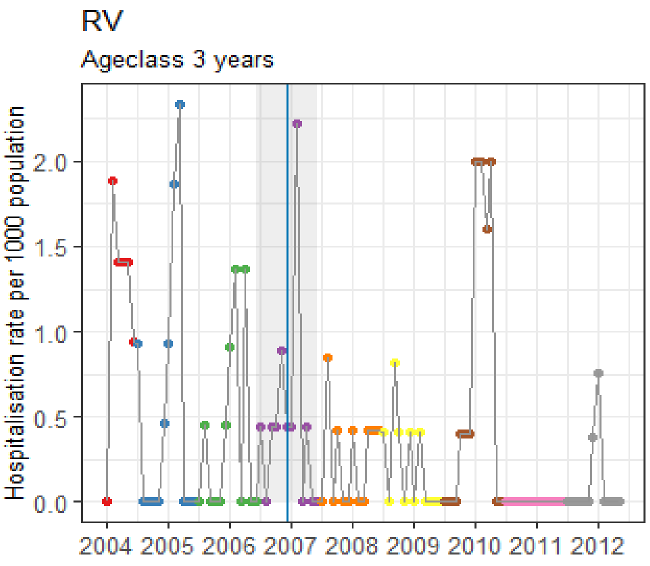

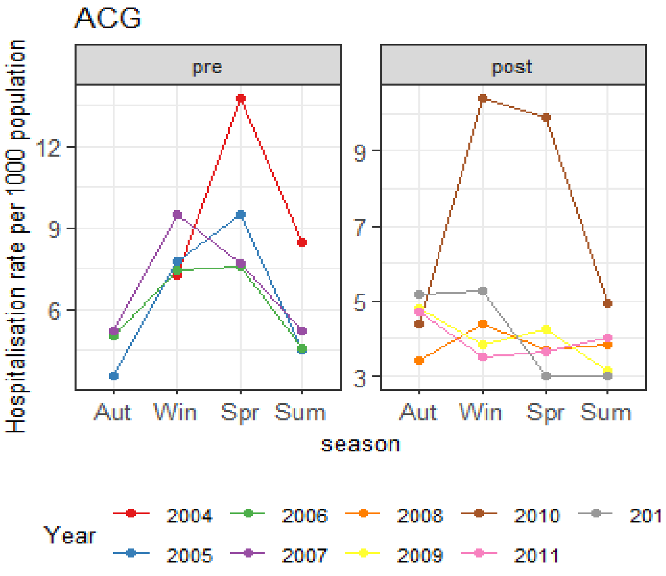

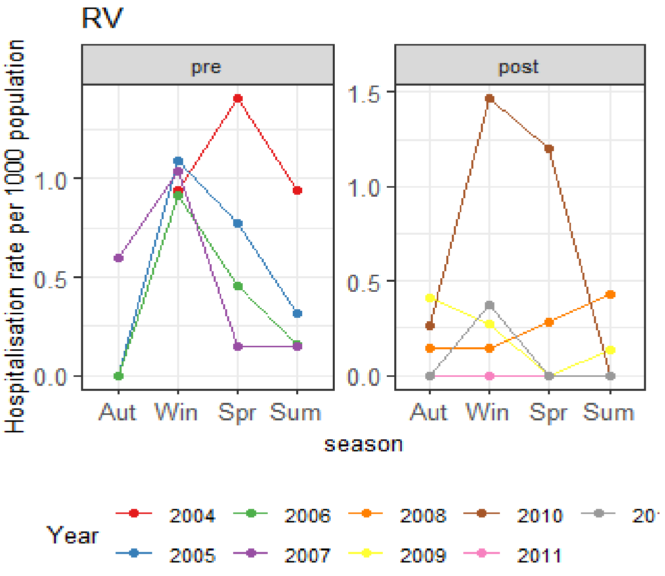

Age class – 4 years

The series are structurally similar to the first age class with level, possible seasonality, and noise:

- Negligible differential stage trends apparent (clear overall trend)
- Change in level not so apparent but still clear
- Change in variability
- Periodicity in the pre-intervention period, not so apparent in the post-rollout

New points of note:

- Sparse data in the RV series
- Peak around 2010 in ACG and RV series
- Insufficient data to model RV

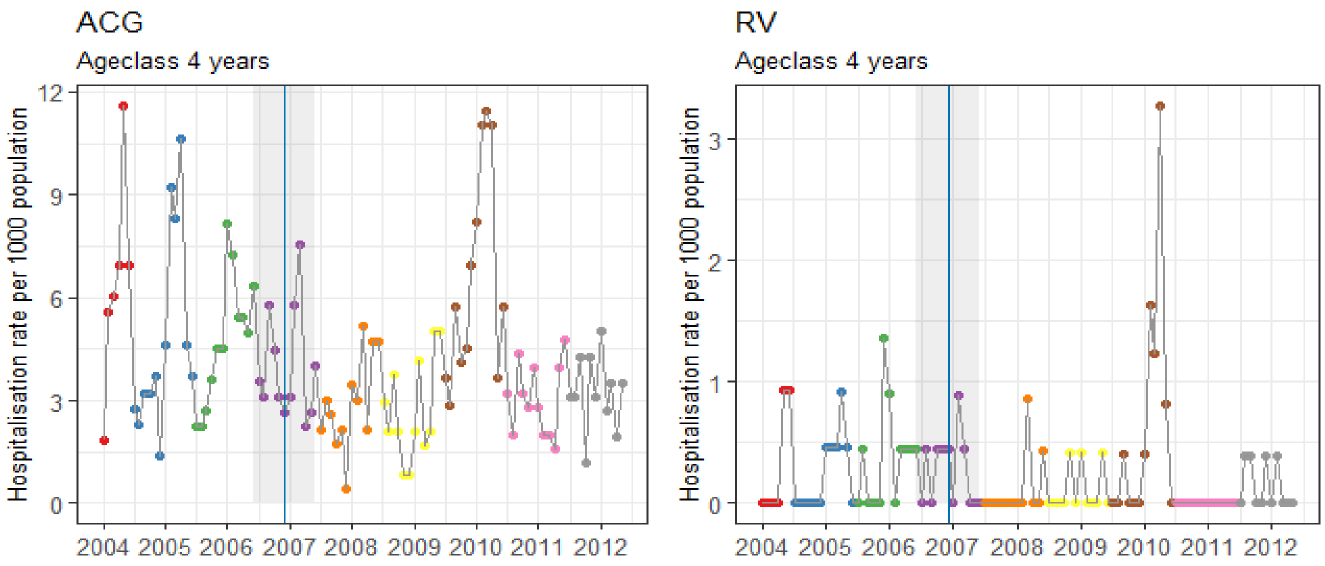

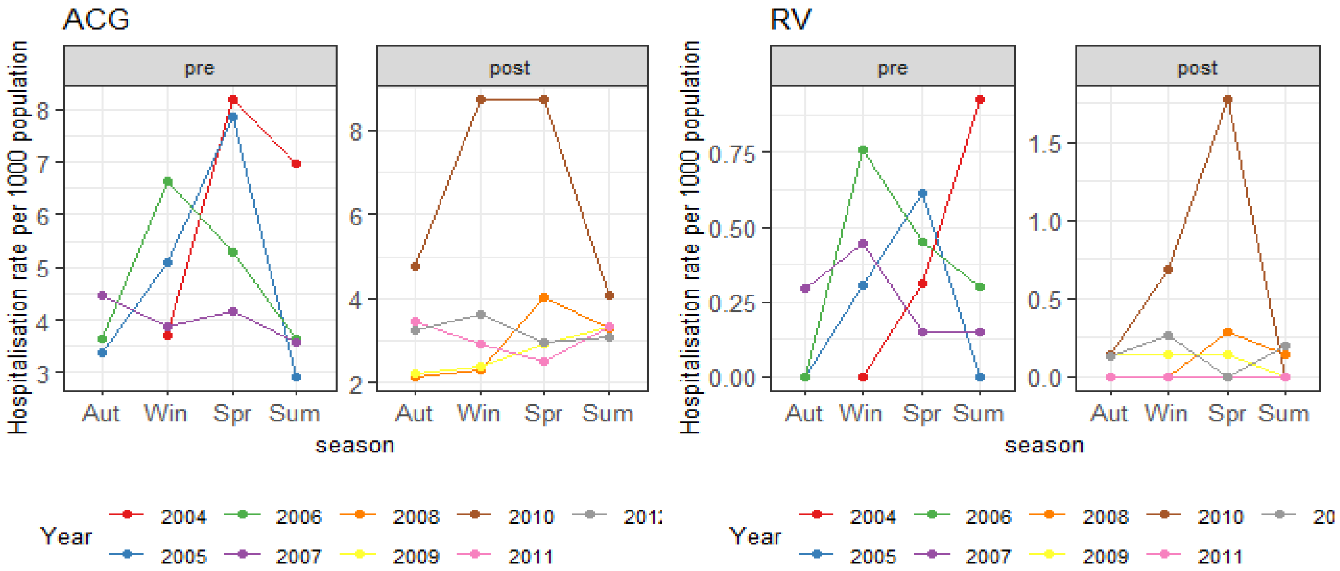

Age class – 5-9 years

Structurally, the series seem to start to change in this cohort relative to the younger cohorts. Points of note:

- Less variability in the month to month rates
- Series looks very much like a random walk
- Temporary drop in ACG followed by peak and then apparent climb in ACG series
- Large number of zeros in the RV series
- Peak around 2010 in ACG and RV series
- Insufficient data to model RV

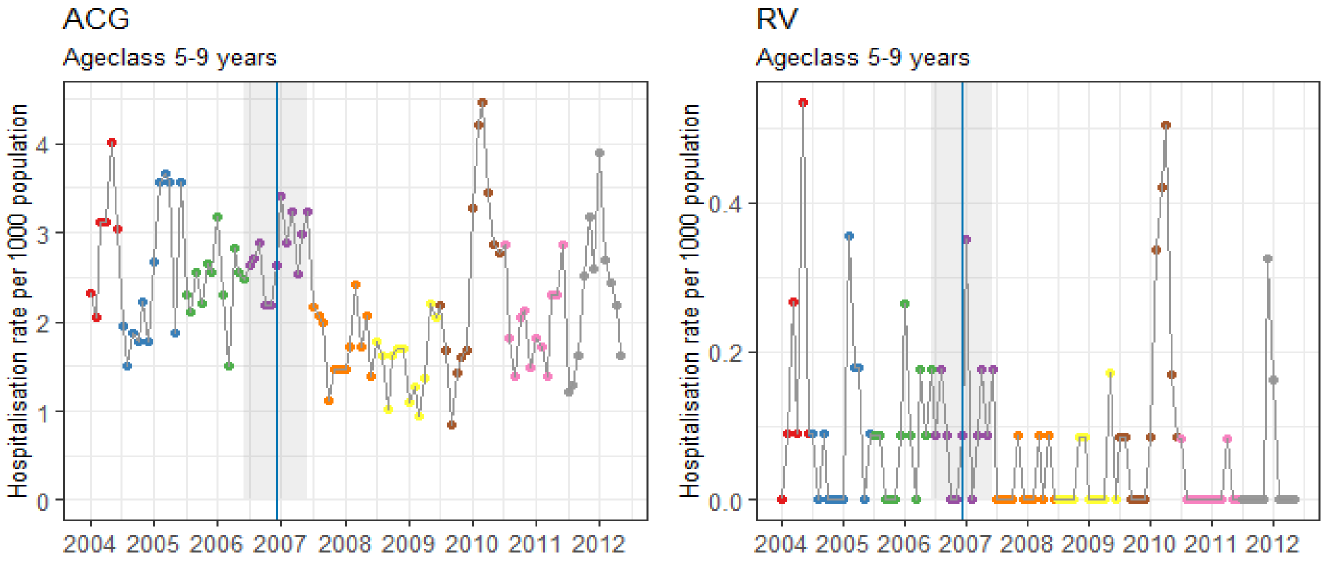

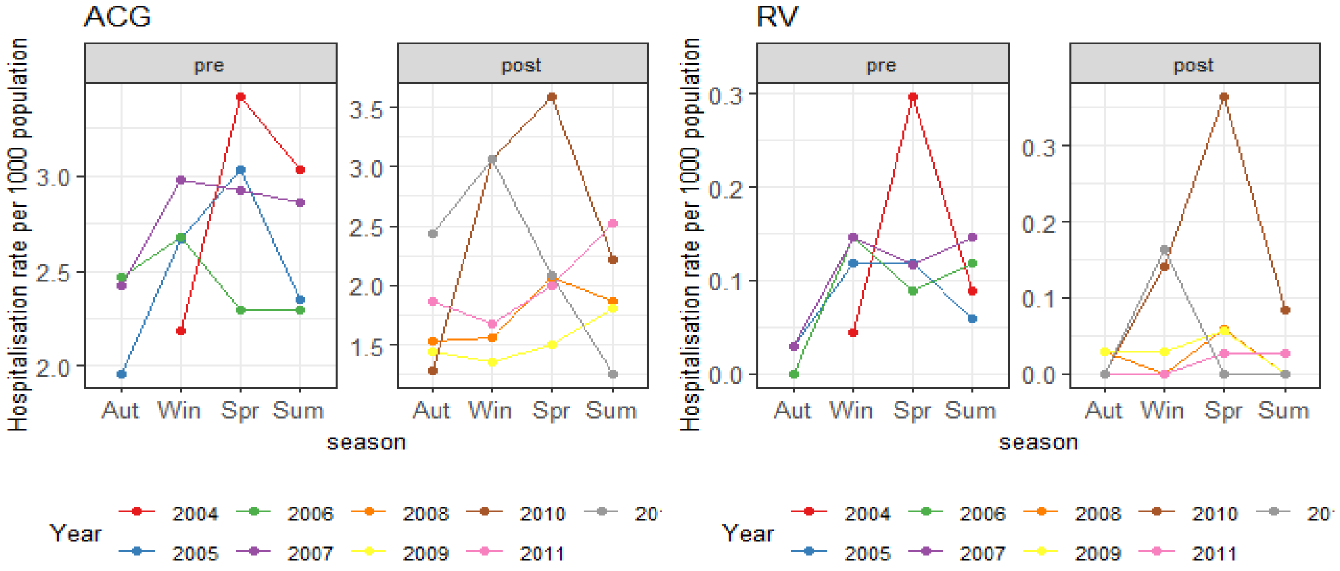

Age class – 10-19 years

The series have structural features of level, possible seasonality, trend, and noise:

- ACG looks flat in pre-intervention.
- ACG post-rollout growth in hospitalisations
- Large number of zeros in the RV series
- Suggestion of peak around 2010 in ACG series but not apparent in RV
- Insufficient data to model RV

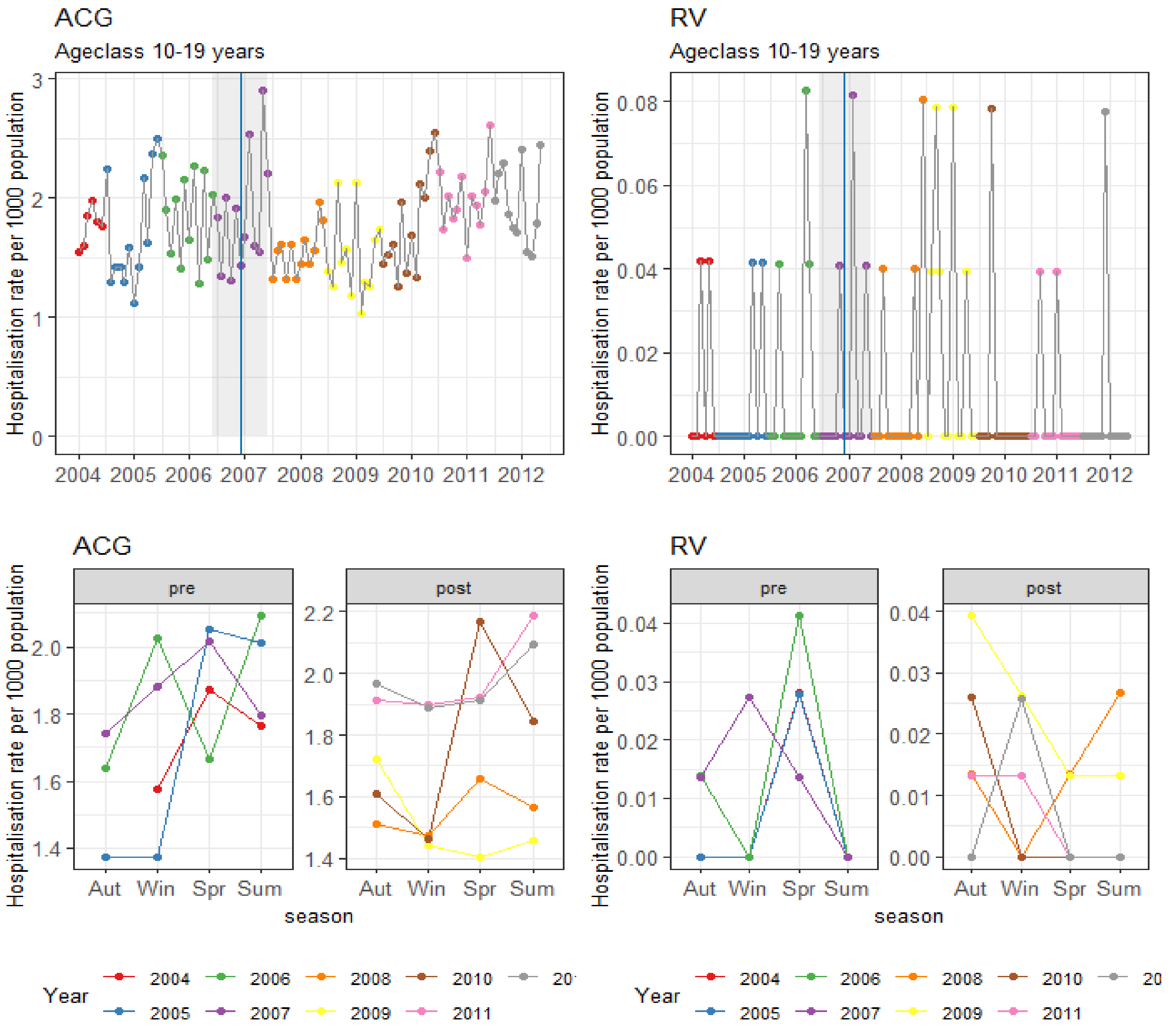

Age class – 20-44 years

The series have structural features of level, possible seasonality, trend, and noise:

- ACG trends in both periods.
- Drop as the post-rollout stage begins in ACG series
- Large number of zeros in the RV series
- Peak around 2010 has completely gone
- Insufficient data to model RV

This could more than likely be adequately modelled as a random walk with drift

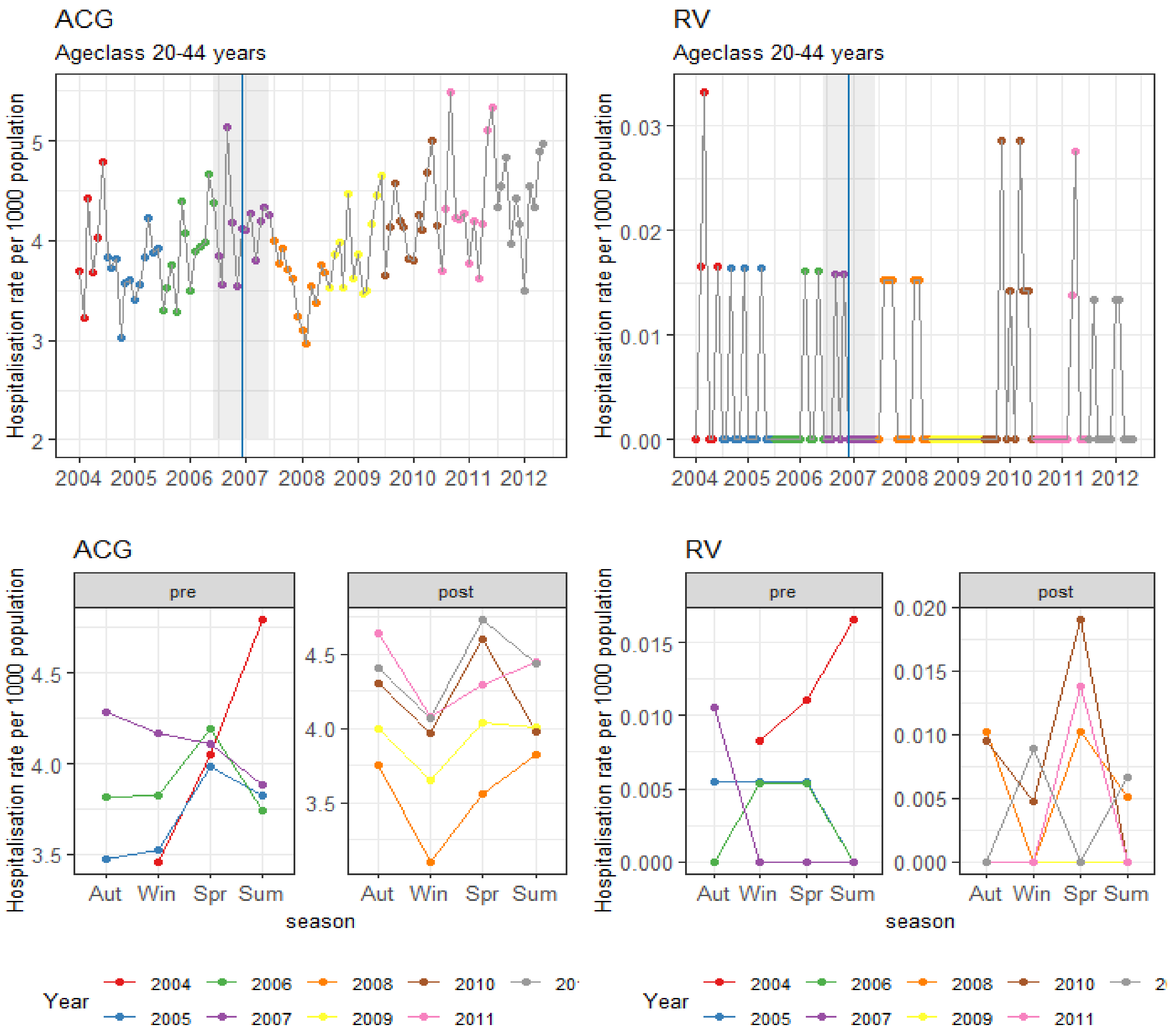

Age class – 45-64 years

The series have structural features of possible seasonality, trend, and noise:

- ACG shows almost seamless trend in both periods.
- Large number of zeros in the RV series
- RV data sparse – insufficient to model
- ACG data look almost negatively autoregressive
- Insufficient data to model RV

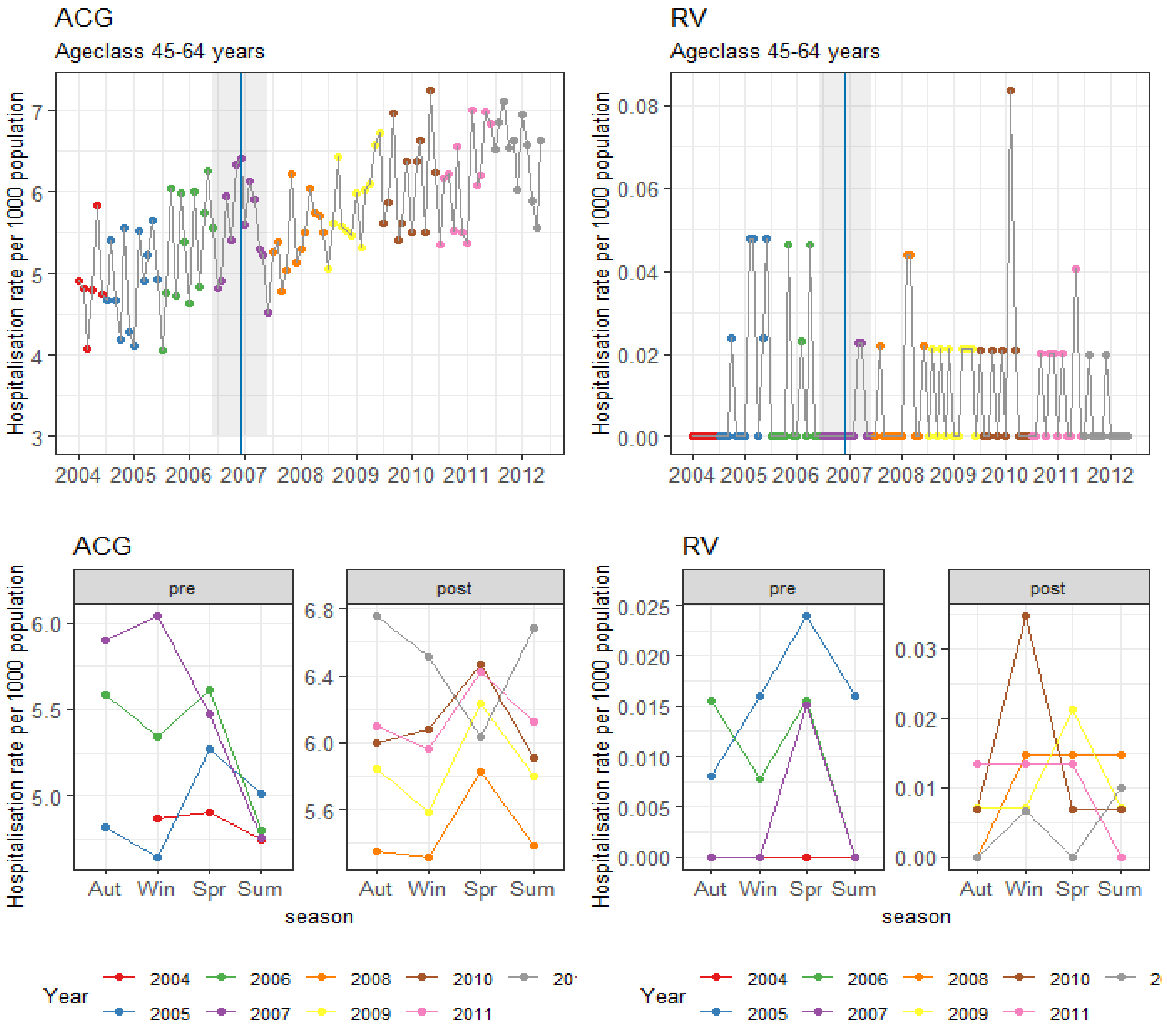

Age class ≥65 years

The series have structural features of possible seasonality, trend, and noise:

- ACG seamless trend in both periods with occasional possible ‘outbreaks’ but could just be stochastic fluctuations
- Large number of zeros in the RV series
- Insufficient data to model RV

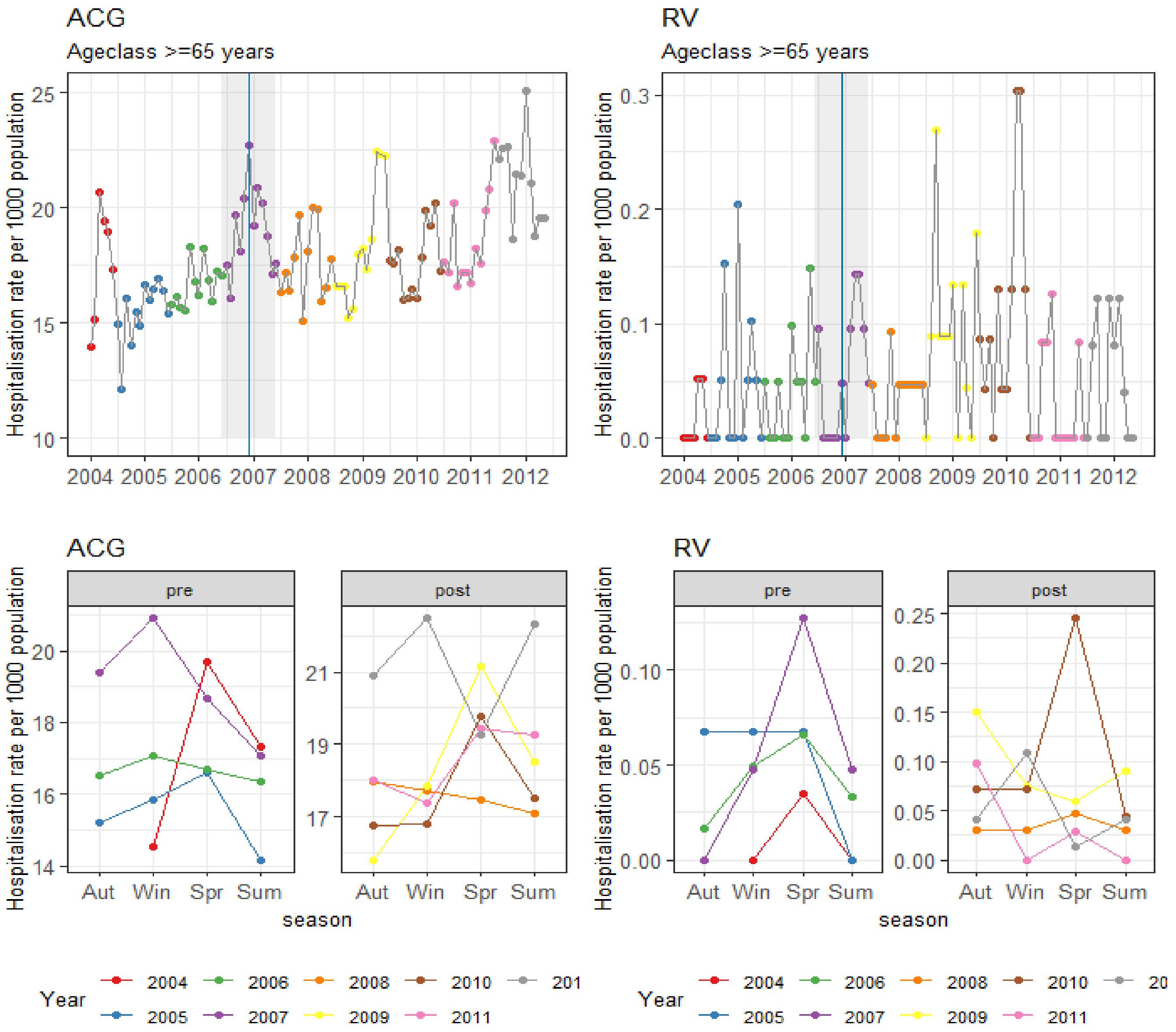

References

1. Bernal JL, Cummins S, Gasparrini A. Interrupted time series regression for the evaluation of public health interventions: a tutorial. International Journal of Epidemiology, 2017, 46(1):348–355
2. Newey WK, West KD (1987), A Simple, Positive Semi-Definite, Heteroskedasticity and Autocorrelation Consistent Covariance Matrix. Econometrica, 55, 703–708.
3. Wagner AK, Soumerai SB, Zhang F, Ross-Degnan D. Segmented regression analysis of interrupted time series studies in medication use research. Journal of Clinical Pharmacy and Therapeutics (2002) 27, 299–309
4. Gelman A, Hill J. Data analysis using regression and multilevel/hierarchical models Cambridge University Press, 2006
5. McCue T, Carruthers EH, Dawe JC, Liu S, Robar A, Johnson K. Evaluation of generalized linear model assumptions using randomisation. Corpus ID: 20141745
6. Dunsmuir WT, Scott DJ. The glarma package for observation-driven time series regression of counts. J Stat Softw. 2015;67(7):1-36.
7. Henley SS, Golden RM, Kashner TM. Statistical modeling methods: challenges and strategies. Biostatistics & Epidemiology. 2020;4(1):105-139
8. Pankratz, A. (1991). Forecasting with Dynamic Regression Models. John Wiley and Sons, New York; Chichester.
